# Host genetics and diet, but not immunoglobulin A expression, converge to shape compositional features of the gut microbiome in an advanced intercross population of mice

**DOI:** 10.1186/s13059-014-0552-6

**Published:** 2014-12-17

**Authors:** Larry J Leamy, Scott A Kelly, Joseph Nietfeldt, Ryan M Legge, Fangrui Ma, Kunjie Hua, Rohita Sinha, Daniel A Peterson, Jens Walter, Andrew K Benson, Daniel Pomp

**Affiliations:** Department of Biological Sciences, University of North Carolina at Charlotte, Charlotte, North Carolina 28223 USA; Department of Zoology, Ohio Wesleyan University, Delaware, Ohio 43015 USA; Department of Genetics, University of North Carolina, Chapel Hill, North Carolina 27599 USA; Department of Food Science and Technology and Core for Applied Genomics and Ecology, University of Nebraska, 329 Food Industry Complex, Lincoln, Nebraska 68583 USA; Department of Pathology, Johns Hopkins University, Baltimore, MD 21205 USA

## Abstract

**Background:**

Individuality in the species composition of the vertebrate gut microbiota is driven by a combination of host and environmental factors that have largely been studied independently. We studied the convergence of these factors in a G_10_ mouse population generated from a cross between two strains to search for quantitative trait loci (QTLs) that affect gut microbiota composition or ileal Immunoglobulin A (IgA) expression in mice fed normal or high-fat diets.

**Results:**

We found 42 microbiota-specific QTLs in 27 different genomic regions that affect the relative abundances of 39 taxa, including four QTL that were shared between this G_10_ population and the population previously studied at G_4_. Several of the G_10_ QTLs show apparent pleiotropy. Eight of these QTLs, including four at the same site on chromosome 9, show significant interaction with diet, implying that diet can modify the effects of some host loci on gut microbiome composition. Utilization patterns of *IghV* variable regions among IgA-specific mRNAs from ileal tissue are affected by 54 significant QTLs, most of which map to a segment of chromosome 12 spanning the *Igh* locus. Despite the effect of genetic variation on *IghV* utilization, we are unable to detect overlapping microbiota and IgA QTLs and there is no significant correlation between IgA variable pattern utilization and the abundance of any of the taxa from the fecal microbiota.

**Conclusions:**

We conclude that host genetics and diet can converge to shape the gut microbiota, but host genetic effects are not manifested through differences in IgA production.

**Electronic supplementary material:**

The online version of this article (doi:10.1186/s13059-014-0552-6) contains supplementary material, which is available to authorized users.

## Background

The mammalian gut harbors a microbiota that consists of hundreds of microbial species whose relative abundances vary considerably among individuals [1-3]. At some extremes of this variation, composition and function of the microbiota show associations with complex diseases and these abnormal microbial assemblages may even contribute to the disease process [4-7]. Despite the growing catalogue of known gut microbes and an increasing understanding of their distributions in populations, the fundamental principles that guide assembly and define structure of the microbiome are largely unknown.

Ecological theory predicts that community assembly is governed by a combination of deterministic, historic, and neutral factors [8]. Evidence now exists that gut microbiota is structured by host-defined deterministic factors specified by the genotype (which relate directly to physiology and immune functions), deterministic environmental factors such as diet, and stochastic factors such as colonization order and history of antibiotic exposure [9]. Though the relative contribution of several of these factors have begun to be estimated individually, systematic studies are needed to understand how these factors converge to shape individualized microbiomes that show stability and resilience.

That natural genetic variation can indeed account for variation in the abundances of taxa of the gut microbiota has been demonstrated in mouse model systems, subsequently leading to the identification of quantitative trait loci (QTL) that affect the relative abundances of specific microbial taxa and groups of taxa in the gut [10-12]. Among the 18 QTLs initially mapped by Benson *et al.* [10], at least three of the microbiota QTL overlapped QTLs for complex diseases, suggesting that genetic predisposition to complex diseases may be attributable, in part, to assembly of abnormal microbiomes. Indeed, variation in several innate response genes is associated with inflammatory and metabolic diseases in humans and these diseases also manifest dysbiosis [13-18]. Although the causal relationships between genetic variation, dysbiosis, and disease are still largely unknown, work in experimental animal models shows that null mutations in innate response genes give rise to dysbiotic microbiota that can bring about disease characteristics when transferred into naïve animals [19-23].

In contrast to innate response genes, it is unclear how genetic variation in adaptive immune genes affects the microbiome. Rag -/- mice, which entirely lack an adaptive immune system, have significant abnormalities in composition of the gut microbiota [24]. However, the innate and adaptive responses have overlapping roles in gut function and innate responses dominate these roles when an adaptive response such as IgA production is abrogated [25-27]. These confounding effects have begun to be untangled, with recent studies showing that signaling through TLR5 can influence immunoglobulin production against flagellar antigens of the gut microbiome [28] and signaling through FoxP3+ T cells plays a role in stimulating IgA production in Peyer’s patches that modulates members of the *Lachnospiraceae* [29].

Though host factors can contribute measurably to fecal microbiota composition, these differences do not appear to explain the majority of the variation contributing to individuality. Thus, environmental and stochastic factors must also play significant roles. Several studies show measurable influences of dietary modulation on gut microbiota composition [7,30-32], with short-term changes in diet resulting in relatively rapid responses in the relative abundances of taxa within the gut microbiota [33]. Even relatively minor short-term changes such as inclusion of whole grains or prebiotic oligosaccharides can translate into significant, albeit temporary, changes in microbiome composition [34,35]. Relationships between microbiome composition and long-term diet are poorly understood but seem to be reversible in mice [36]. Nonetheless, some associations of long-term diet with overall microbiota composition have been reported in humans [37], making it still unclear if diet on its own is a significant contributor to the individuality of the gut microbiota.

Collectively, each of these deterministic factors (diet, immune function, and host genotype) can have measurable effects when studied independently, but it is unknown how these factors converge to ultimately shape composition of the microbiota. To provide insight into the interactions of these factors, we conducted a genome scan to search for QTL controlling composition of the microbiota and QTL controlling variable region utilization among expressed IgA in a mouse intercross model with a dietary variable (high-fat versus conventional diet). The mouse population was developed as an advanced intercross population produced from crosses of mice with a genetic predisposition to dietary-induced obesity (C57BL/6J) with those in a strain selected for high voluntary wheel running. At weaning, the population was randomly assigned to normal or high-fat diets for 6 to 8 weeks and sampled for microbiota composition with tissue from the ileum of the same animals sampled at necropsy for RNA extraction and measurement of mRNA from expressed IgA.

## Results

### Basic statistics and variance components of the generation 10 microbiota

As we have reported previously [10], a large proportion of the taxa detected by pyrosequencing show a sparse distribution across the animal population; and of the 472 mice in this G_10_ population of mice, 203 taxa (OTUs at 97%) were detected in at least 75% of the animals. The mean relative abundances of these 203 consistently-detected taxa across all animals were quite broad, in the range of 0.045 for dominant taxa such as *Alistipes* OTU15 to 0.00027 for low abundance taxa such as OTU76601. There was also little relationship between the mean abundance of taxa and the range as some dominant taxa such as *Parabacteroides* OTU3 ranged nearly 1,000-fold across the animals (from abundances of 0.222 to 0.000226) while some lower abundance taxa such as *Odoribacter* OTU1 showed a tighter distribution (abundances of 0.006 to 0.00011). For statistical analyses, the relative abundances were log-transformed to reduce the effects of skewness, and the means and standard deviations of these log-transformed abundances are given in Additional file 1.

Estimates of the variance components (Additional file 2) for the microbiota taxa abundances vary considerably among the 203 taxa. Differences among the cohorts account for an average of 9.7% of the total variation, although these percentage values are in the range of 0 (in 7 taxa) to as much as 43.2%. Contributions from family differences average about one-half of that for cohort (4.8%), with 49 taxa showing no differences. Litter differences contribute on average 6.1% to the total variation, although again with a number of taxa (N = 29) showing no differences between litters. Residual variation contributes by far the largest amount to the total variation, averaging 79.4% and varying from 43.8% to 97.9% among the 203 taxa. Thus excluding the environmental cohort and litter contributions, an unknown fraction of the remaining 84.2% contributed by family and residual differences is genetic in origin.

### Compositional features of the G_4_ and G_10_ gut microbiota

**Figure 1:**
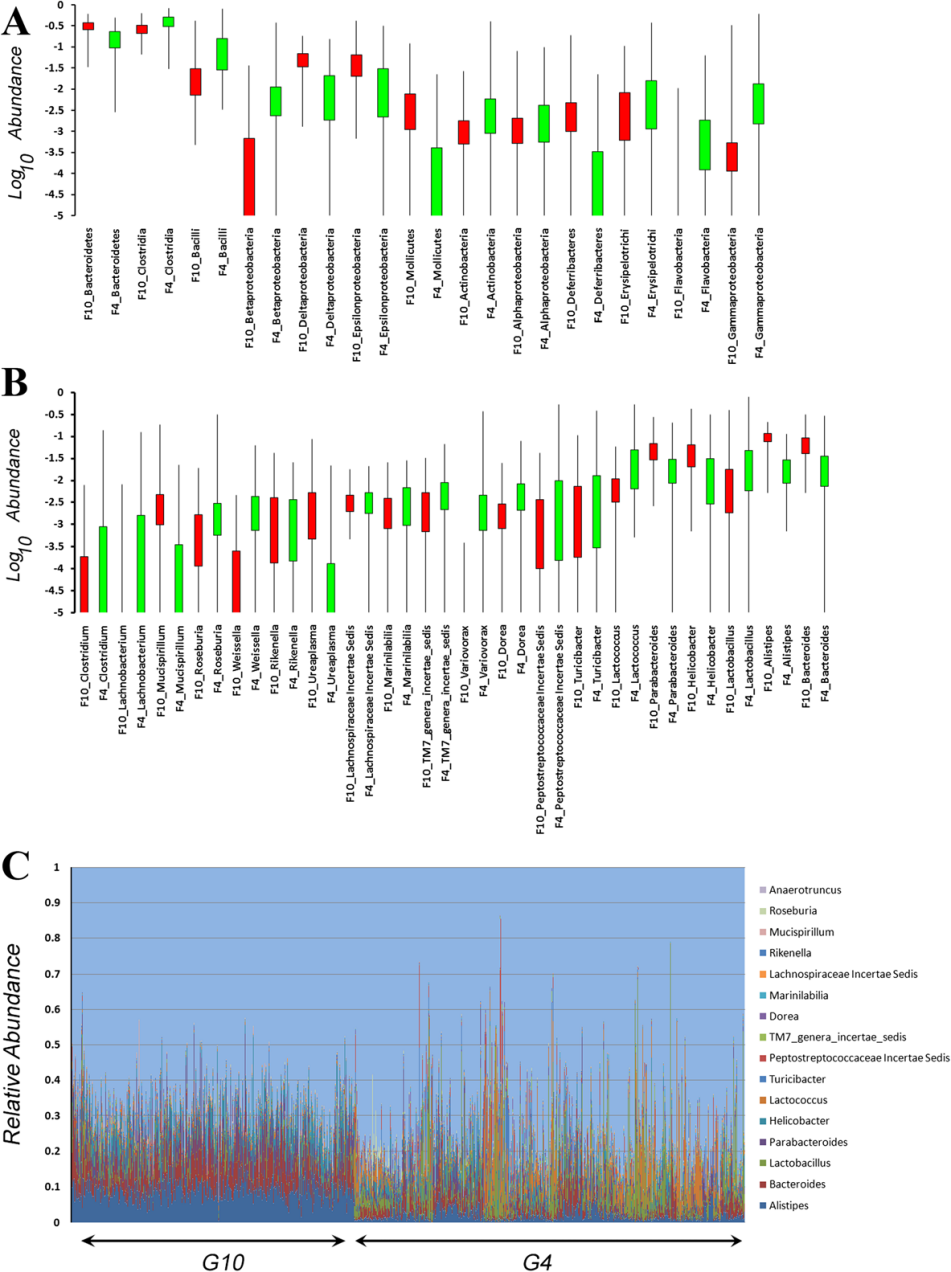
**Comparison of microbiota composition between G**_**4**_ **and G**_**10**_ **populations. (A, B)** Box and whisker plots for the Log_10_ relative abundances of taxa (**(A)** Class, **(B)** Genus) that are shared between the G_4_ (green) and G_10_ (red) populations. The boxes represent 75% of the data and whiskers indicate the range. **(C)** The relative abundances of the 16 shared genera between the G_4_ and G_10_ populations. G_10_ mice are shown on the left by cohort and G_4_ mice are on the right ordered by cohort.

The G_10_ population showed several major differences in composition of the microbiota when compared to the population mapped at G_4_ [10], many of which could be observed even at high taxonomic ranks. As illustrated in Figure 1, the G_10_ population had significantly higher levels of taxa belonging to the *Bacteriodetes*, Delta *Proteobacteria*, Epsilon *Proteobacteria*, *Mollicutes*, and *Deferribacteres*. This was offset by decreased levels of *Clostridia*, *Bacilli*, Beta *Proteobacteria*, Gamma *Proteobacteria*, and *Flavobacteria*. This same pattern could also be detected at the genus level (Figure 1B), with the G_10_ mice showing substantial elevation in members of the *Bacteriodetes* (*Bacteriodes*, *Parabacteriodes*, *Rikenella*, *Allistipes*), Epsilon *Proteobacteria* (*Helicobacter*), Delta *Proteobacteria* (*Mucispirillum*), and *Mollicutes* (*Ureaplasma*) that are offset by decreases in members of the *Clostridia* (*Lachnobacterium*, *Roseburia*, *Dorea*) and *Bacilli* (*Lactobacillus*, *Lactococcus*, *Weissella*), and Beta *Proteobacteria* (Variovorax). Phylogeny-based analysis of the 200 most abundant OTUs from a random selection of 100,000 pooled sequences of the G_4_ and the G_10_ animals (balanced for cohort in G_4_ and G_10_ and diet in G_10_) also showed many of these same differences (Additional file 3), with expansion of the diversity in taxa attributable to the *Bacteridetes* that was offset by a reduction in diversity of taxa attributable to the *Firmicutes* and the *Proteobacteria*. Estimates of alpha diversity using these same 100,000 sequences from the G_4_ and G_10_ populations (based on Shannon and Inverse Simpson indices) showed slightly higher diversity in the G_4_ animals, but the differences were not statistically significant (*P* <0.09). Thus, despite the dramatic changes in taxonomic composition of the microbiota between generations G_4_ and G_10_, there was little change in the overall levels of diversity.

Even though compositional differences in the microbiota emerged at G_10_, several general features were still conserved in the G_4_ and G_10_ populations. In both studies, only a small portion of the taxa were measurable across a significant proportion (75%) of the mice. Because genus-level processing of the pyrosequencing data by the CLASSIFIER algorithm is common to both studies, we examined the genera comprising the Core Measurable Microbiota (CMM) in both studies. All of the 16 genera of the G_10_ CMM were found among the 19 genera comprising the G_4_ CMM. Collectively, these 16 CMM genera comprise 40% to 50% of the total microbiota across all mice (Figure 1C). Though shared, these 16 CMM were distributed quite differently in the G_4_ and G_10_ populations. For example, members of the genera *Alistipes*, *Bacteriodes*, and *Parabacteriodes* dominate nearly all of the G_10_ mice but are only dominant in groups of G_4_ mice corresponding to individual cohorts.

### Effect of high-fat diet on the G10 microbiota

**Figure 2:**
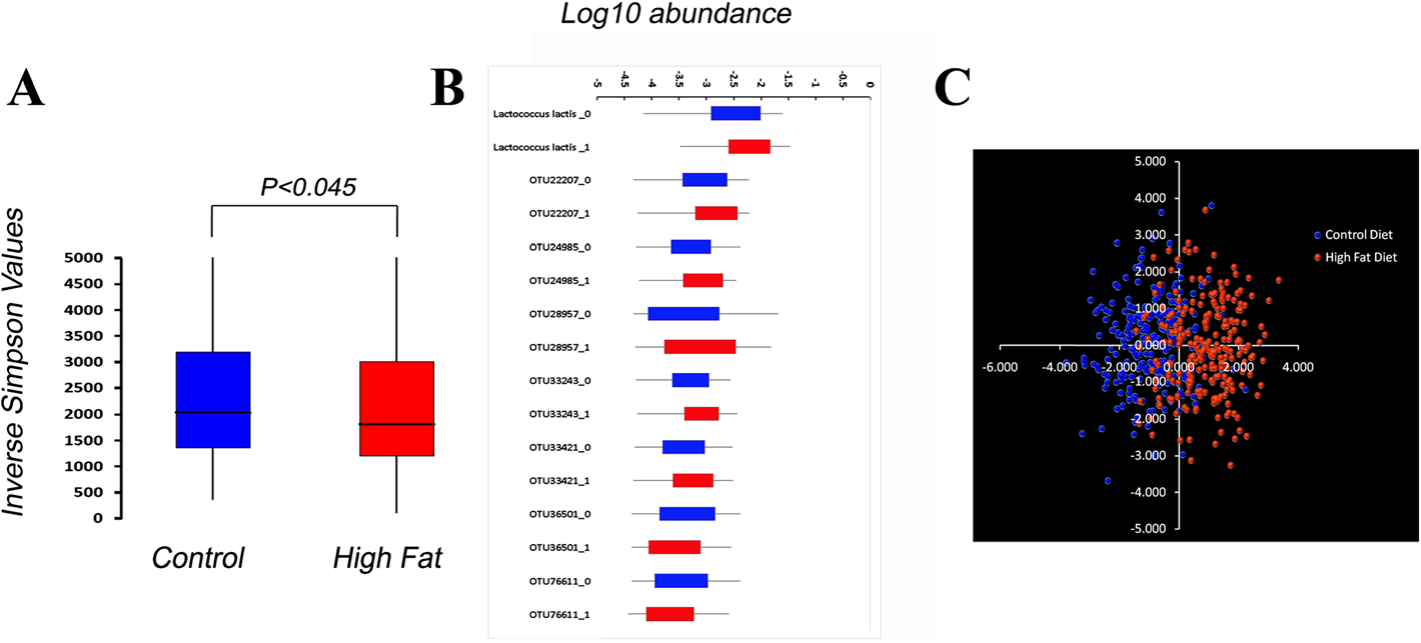
**Effect of diet on the G**_**10**_ **microbiota. (A)** Box and whisker plots of the Inverse Simpson’s index for animals on the control or high-fat diet. The microbiota from animals fed the control (blue) or high-fat (red) diets show a significant effect of diet (one-sided *t*-test: *P* <0.045). **(B)** The distributions of eight taxa showing a statistically significant effect of diet (Bonferonni-adjusted *P* <0.00000483) are depicted in box and whisker plots. The boxes define 75% of the data points. Blue = control diet, Red = high-fat diet. **(C)** The log-transformed abundances of 34 taxa with the smallest, non-adjusted ANOVA *P* values were used as variables for Linear Discriminant Analysis (LDA) with diet and parity as factors. The first two LDA functions account for >60% (X axis) and >30% (Y-axis) of the variation in these taxa.

To examine the effects of diet on compositional features of the microbiota, we first compared estimates of alpha diversity in the microbiota across animals fed control or high-fat diets. As shown in Figure 2A, the inverse Simpson’s index (1/D) showed modest, but statistically significant differences between the animals fed control versus high-fat diets, with animals on the high-fat diet displaying reduced levels of diversity. ANOVA identified 54 taxa showing significant effect of diet (*P* <0.05) but the Bonferonni-corrected significance level (*P* <0.00000483), left only eight of these 54 taxa passing the stringent multiple-testing threshold for significance. Even these eight taxa showed only modest differences in their distributions (Figure 2B). Likewise, Linear Discriminant Analysis (LDA) of the log-transformed abundances of the 34 taxa with smallest *P* values (from ANOVA) also showed a small, but measurable effect of diet (Figure 2C), with microbiota from animals fed control or high-fat diet displaying partial separation, almost exclusively in the first (X-axis) dimension.

Collectively, despite the difference in fat content of the diets, diet-based differentiation of the microbiota in our population was minimal and likely due to the cumulative effects of small differences across multiple taxa, as opposed to large shifts in small numbers of taxa.

### QTLs affecting relative abundances of G_10_ gut microbial taxa

Table 1 gives a summary of all QTLs affecting the relative abundances of taxa from the gut microbiota of the G_10_ mice. Over all 203 taxa, a total of 42 QTLs were discovered, including 22 that had LOD scores reaching the 5% genomewide level of significance. Their confidence intervals average 9.85 Mb with a standard deviation of 5.25 Mb. These QTLs affect 39 of the 203 different taxa (19%), 36 of which are affected by a single QTL. Three taxa, OTU29627, OTU17740, and OTU30840, each are affected by two QTLs on different chromosomes. Using a strict approach to correct for multiple testing, FDR values were calculated from the probabilities associated with the LOD scores of the G_10_ QTLs (Table 1), and these are in the range of 0.002 to 0.513. Only a single QTL on Chr 9, which controls the abundance of the *Alistipes* OTU41353, exceeds this strict experiment-wide tthreshold. Overall, the FDR procedure suggests roughly one-half of the 42 total G_10_ QTLs could represent false positive results.

**Table 1:** **QTL statistics for the microbiome traits in the G**_**10**_ **mouse population**

**Trait**	**Best hit (genus)**	**Ch**	**Position (Mb)**	**CI (Mb)**	**LOD**	**FDR**	***a***	***d***	**%**	**Interaction**
OTU15028	Blautia	1	49.1	43.3-50.7	4.53*	0.404	**-0.20**	**-0.15**	4.72	Diet (C)
*Butyricicoccus*		1	127.2	123.3-140.7	3.97	0.507	**-0.18**	0.02	4.50	Diet (HF)
*ButyricicoccusOTU7*		1	127.2	123.3-140.7	4.30*	0.425	**-0.18**	0.03	4.81	Diet (HF)
OTU18932	Prevotella	1	173.3	167.0-182.1	4.29*	0.425	**0.09**	0.05	4.51	
*ParabacteroidesOTU6*		2	59.5	57.5-62.5	4.08	0.431	**0.27**	0.04	5.40	
OTU24985	Alistipes	2	60.2	57.5-62.5	4.21	0.469	0	**0.15**	4.01	
OTU29627	Hydrogenoanaerobacterium	2	172.5	170.3-173.6	4.51*	0.404	0.01	**-0.24**	5.48	Sex (M)
		9	29.8	22.0-33.4	4.51*	0.404	**-0.16**	-0.02	4.71	
OTU23089	Clostridium	3	16.9	11.8-22.6	4.05	0.431	**0.19**	**-0.21**	3.49	
OTU26092	Blautia	3	20.4	11.8-22.6	4.97*	0.356	**0.16**	**-0.25**	3.87	
OTU17889	Roseburia	4	12.5	8.3-19.7	3.99	0.480	**0.11**	**0.18**	3.60	Diet (C)
OTU3615	Desulfotomaculum	5	115.2	112.9-118.1	4.07	0.431	**-0.24**	**-0.28**	3.82	
OTU32093	Eubacterium	6	54.0	53.1-59.2	5.08*	0.356	0.04	**0.22**	5.20	
OTU35558	Desulfocurvus	6	115.6	112.2-120.0	4.42*	0.469	**0.09**	**-0.08**	4.52	
*Lactobacillusjohnsonii*		6	126.9	122.4-131.6	4.08	0.431	**-0.30**	-0.11	4.12	
*ATCC33200*										
OTU14099	Butyricimonas	8	71.6	64.4-79.1	4.66*	0.404	**-0.11**	**-0.27**	4.62	
OTU16090	Alistipes	8	74.9	64.4-79.1	4.10	0.439	**-0.15**	**-0.25**	4.20	
OTU27257	Alistipes	8	77.5	66.5-79.1	4.16	0.431	**-0.11**	**-0.25**	4.08	
OTU41353	Alistipes	9	37.3	34.6-40.9	7.99*	0.002	**-0.16**	-0.04	8.29	
OTU29084	Clostridium	9	40.2	33.5-42.3	4.56*	0.404	**-0.11**	0.06	4.79	
OTU17740	Prevotella	9	40.7	33.5-40.9	5.87*	0.139	**-0.26**	0.07	5.21	Diet (C)
		X	66.1	54.5-74.6	4.07	0.469	**-0.18**	-0.02	5.21	
OTU25269	Odoribacter	9	40.7	33.5-40.9	6.01*	0.137	**-0.26**	0.02	6.63	Diet (C)
OTU13989	Bacteriodes	9	40.7	40.5-42.4	4.51*	0.342	**-0.10**	**0.13**	4.20	Diet (HF)
OTU25483	Bacteriodes	9	40.7	33.5-40.9	6.04*	0.137	**-0.21**	0	6.83	Diet (C)
*Lactococcus lactis*		9	113.3	112.3-115.0	3.95	0.513	**0.11**	-0.07	3.71	
OTU21572	Roseburia	10	6.4	4.7-9.07	4.24	0.469	0.07	**0.17**	3.72	
OTU33466	Oscillibacter	11	41.8	35.1-44.5	4.12	0.469	**-0.15**	**0.10**	4.29	
OTU22207	Alistipes	11	97.8	93.4-114.0	3.97	0.463	0.05	**0.11**	3.31	
*Odoribacter*		14	17.1	10.5-20.3	4.39*	0.404	**-0.04**	**-0.11**	3.26	
*OdoribacterOTU6*		14	17.1	10.5-20.3	4.04	0.431	**-0.04**	**-0.10**	3.04	
OTU30840	Clostridium	14	71.1	67.6-87.5	4.67*	0.404	0.01	**-0.25**	6.40	
		18	68.4	65.4-70.2	3.93	0.500	-0.02	**0.22**	4.16	
OTU46742	Hydrogenoanaerobacterium	14	88.7	79.7-87.5	4.87*	0.404	**-0.11**	**-0.13**	4.82	
OTU20360	Bacteriodes	16	6.9	3.98-9.92	4.28*	0.425	**-0.12**	**0.08**	4.52	
*AlistipesOTU15*		16	44.8	42.7-48.1	4.21	0.469	**-0.06**	**0.07**	4.76	Sex (M)
*Mucispirillum*		16	45.7	42.7-57.8	4.72*	0.404	**0.28**	**-0.26**	6.54	
*Mucispirillumschaedleri(T)*		16	45.7	42.7-57.9	4.72*	0.404	**0.28**	**-0.26**	6.54	
*Lactobacillus*		16	63.3	51.6-70.4	4.27*	0.469	-0.10	**0.31**	3.77	
OTU17491	Odoribacter	17	48.7	51.9-58.2	4.11	0.439	**-0.09**	**0.12**	2.99	
OTU23606	Bacteriodes	18	83.1	83.1-	4.01	0.480	0	**-0.21**	4.03	
OTU29519	Clostridium	19	24.3	22.6-24.7	4.04	0.476	**-0.22**	**0.23**	4.30	

Shown are all QTLs affecting the microbiota traits that had LOD scores reaching the suggestive or significant (*) threshold level of significance. The false discovery rate (FDR) also is listed for each QTL, indicating its probability of being a false positive result. Locations and confidence intervals of the QTLs are given in Mb (from NCBI Build 37). Also shown is the percentage contribution (%) of each QTL to the total variance of each trait, and its additive (*a*), dominance (*d*) genotypic effects (bolded values indicate significance). QTLs affecting the microbiota in males only = Sex (M) and in females only = Sex (F). QTLs affecting the microbiota in mice fed only the control diet = Diet (C) and for mice fed only the high-fat diet = Diet (HF).

If the strict experiment-wise threshold is relaxed, multiple examples of overlap are observed (Table 1) among the genomic sites of significant (genome-wide *P* <0.05) and suggestive (genome-wide *P* <0.1) QTLs. Such overlap implies pleiotropy and underlying covariation of microbial taxa. The most obvious example of this is seen for six QTLs on Chr 9 at 37.3 Mb to 40.7 Mb that each affects a different taxon. All six of these QTLs, especially the four mapping to 40.7 Mb, may represent a single gene or set of closely linked genes with independent effects on these traits. Notably, these six QTLs have the lowest FDR values and thus the highest probability of being true positives. The traits affected by these six QTLs show three different patterns of covariation across the animals, suggesting alleles from two or more closely linked genes may be contributing to the phenotypic segregation. Altogether, 27 of the 42 total QTLs have non-overlapping confidence intervals, suggesting that there may be as few as 27 unique detectable QTLs affecting these traits. Over half (N = 19) of the 27 unique QTLs affect only one trait, however, so putative pleiotropy is not extensive.

Thirty-three of the 42 microbiota QTLs exhibit significant additive effects with an average absolute *a* value of 0.161. Of these 33, most (24) are negative in sign, indicating that the HR allele at these loci generally acts to increase the abundance of the affected taxa. The number of QTLs showing significant dominance genotypic effects is 29, nearly as many as those exhibiting additive genotype effects. Further, the mean of the absolute values of these significant dominance effects (*d* values) is 0.186, slightly greater than that for the additive effects. The *d*/*a* ratios (not shown) suggest that 13 of these 39 QTLs show dominance whereas 10 exhibit overdominance and six exhibit underdominance. An example of overdominance (heterozygote greater than either homozygote) is shown by the QTL on Chr 6 (54 Mb) affecting OTU32093 with a dominance value of 0.22 that is over five times greater than its additive value of 0.04.

The percentage of the total phenotypic variation explained by the microbiota QTLs is in the range of about 3% to over 8%, averaging 4.6%. The highest percentages explained are seen for the QTL on Chr 9 mentioned above although a QTL on Chr 16 affecting *Mucispirillum* (and *Mucispirillium schaedleri* which accounts for most of the *Mucispirllum*), and one on Chr 14 affecting OTU30840, account for over 6% of the variation in their abundances. In the G_4_ population, the percentage contributions of the microbiota QTLs were quite comparable, varying from 1.5% to 9.0% and averaging 4.7% [10].

### QTL replication in the G_10_ population

**Figure 3:**
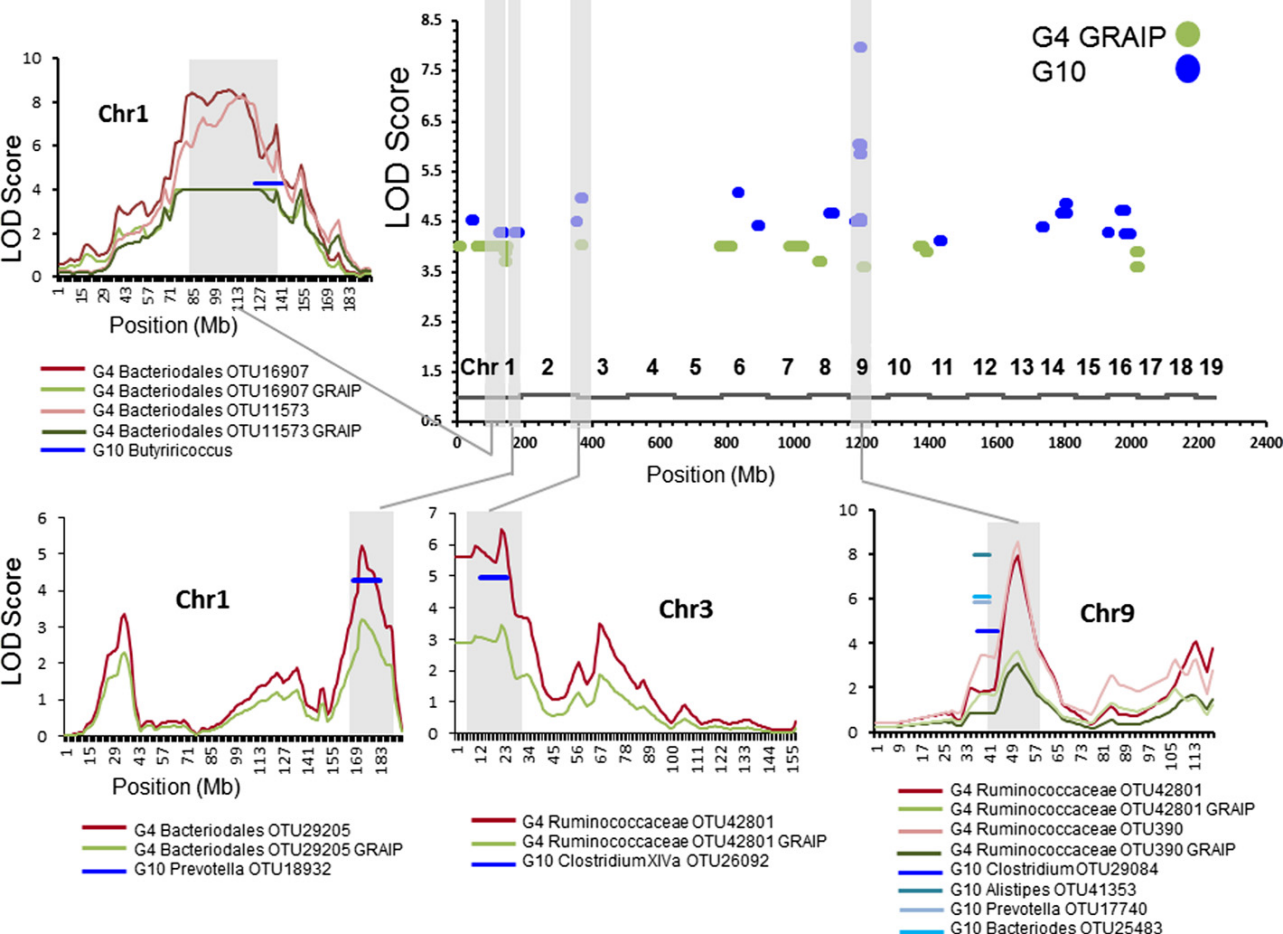
**Relative positions of QTLs controlling gut microbial taxa from the G**_**4**_ **and G**_**10**_ **populations.** In the graph on the top right (Whole Genome), peak positions of the QTLs from the G_4_ (green dots) and G_10_ (blue dots) generations are plotted along the length of the 19 mouse autosomes. The relative positions of each chromosome are indicated above the X axis. Regions where confidence intervals overlap from G_4_ and G_10_ QTLs are highlighted in gray and are plotted in the corresponding graphs to the left and below. Plots for the overlaps on the individual chromosomes show the chromosome positions (X axis) and naïve/GRAIP-adjusted LOD scores (Y axis) for the significant G_4_ QTLs with the confidence intervals of the G_10_ QTLs plotted as a single line at its corresponding position and peak LOD score. Individual taxa are color-coded below each graph.

The high number of potentially false positive QTLs from multiple testing led us to search for validation through potentially overlapping QTLs mapped previously in the G_4_ population. The initial comparison revealed no overlap, but given the differences in taxonomic composition and given that the G_4_ QTLs were originally mapped only at the taxonomic rank of genus and higher, it seemed possible that the lack of overlap was partially due to the different levels of taxonomic resolution used for mapping. To overcome this confounder, the taxonomic resolution was normalized by processing the G_4_ microbiota data set using the same OTU pipeline used for the G_10_ data. This generated 331 species-level OTUs and 23 genera (Additional file 4) that met our trait distribution threshold (at least 5 reads per taxon across 75% of the animals). These taxa were then mapped using the G_4_ genotyping data as done previously [10] with the robust permutation-based thresholds of the GRAIP algorithm to account for structural relatedness among families [38]. A total of 21 significant QTLs were detected among the G_4_ OTUs (Additional file 5) and with equivalent levels of taxonomic resolution, four of these G_4_ QTLs now shared overlapping peaks with G_10_ QTLs or were immediately adjacent to a G_10_ QTL (Figure 3). These included two different G_4_ QTLs on Chr 1, and one each on Chr 3 and Chr 9. Notably, the G_10_ QTLs on Chr 9 had the highest degree of statistical support. In addition to overlapping peaks, three of these four QTLs affect organisms that are taxonomically related in the G_4_ and G_10_ animals. For example, the G_4_/G_10_ QTLs around 170 Mb of Chr 1 control OTUs belonging to the genera *Bacteriodes* (G_4_) and *Prevotella* (G_10_), both of which belong to the taxonomic order *Bacteriodales*. Likewise, the QTL peaks on Chr 3 and Chr 9 control OTUs belonging to the order *Clostridiales* (a member of the family *Ruminococcaceae* in the G_4_ population and an OTU belonging to the genus *Clostridium* in the G_10_ population). These overlapping QTLs controlling taxonomically related organisms in separate populations are strongly suggestive of replicated QTLs. In addition, the capacity of these QTLs to influence distinct, but taxonomically related organisms, further illustrates how some host genomic loci can exert pleiotropic effects across cross-sections of phylogenetic space in the microbiome.

### QTL interactions in the G_10_ population

QTLs were tested for potential interactions with sex and with diet by calculating the -2 ln (likelihood) for a model containing all terms, but with the interactions of the *a* and *d* effects with sex or diet. Likelihoods obtained from this model were then compared with the null model lacking the interaction terms. Differences between likelihoods from the two models were further evaluated by chi-square tests. Using a probability cutoff of *P* <0.05 for significance, two microbiota QTLs in the G_10_ population showed significant interactions with sex, and eight QTLs interacted with diet (Table 1). The sex interactions involve QTLs on Chr 2 (at 172.5 Mb) and Chr 16 (at 44.8 Mb), and in both cases, significant effects were seen only in the male mice. Among the 8 QTLs showing interactions with the dietary environment, only four separate genomic sites are represented. However, despite the small number of loci influenced by diet, we note that some of these loci are quite complex.

**Figure 4:**
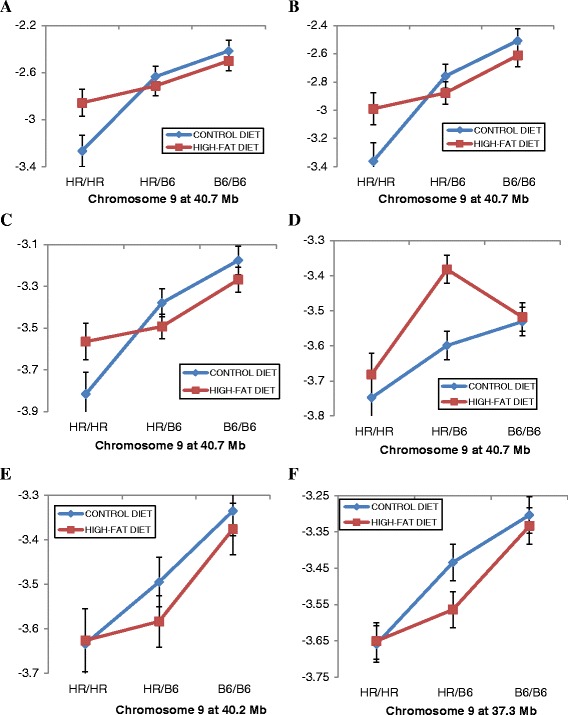
**QTL on Chr 9 show complex interactions with diet. (A-F)** Plots of genotypic values at the QTL peak (X axis) are shown for six different OTUs that have peaks at the same or similar location on Chr 9 and which show differential effects depending on whether the G_10_ mice were fed the control or the high-fat diet. Relative abundances of the OTUs for animals having the HR/HR parental, HR/B6 heterozygote, or B6/B6 parental genotypes at QTL peak are plotted on the Y-axis. **(A)** OTU17740. **(B)** OTU25269. **(C)** OTU25483. **(D)** OTU13989. **(E)** OTU29084. **(F)** OTU41353. Error bars based on standard error.

Particularly noticeable is the set of four QTLs mapping to the same position on Chr 9 that show different effects depending on the dietary environment (Figure 4). QTLs for OTU17740 (Figure 4A), OTU25269 (Figure 4B), and OTU25438 (Figure 4C) show significant QTL effects only in mice fed the control diet while the QTL for OTU13989 (Figure 4D) shows significant effects only for mice fed the high-fat diet. Not surprisingly, the abundances of OTU17740, OTU25269, and OTU25438 show high degrees of correlation across the G_10_ mice but no correlation with OTU13989. The QTLs for OTU29084 (Figure 4E) and OTU41353 (Figure 4F), which also map to a similar position, show no interaction with diet.

**Figure 5:**
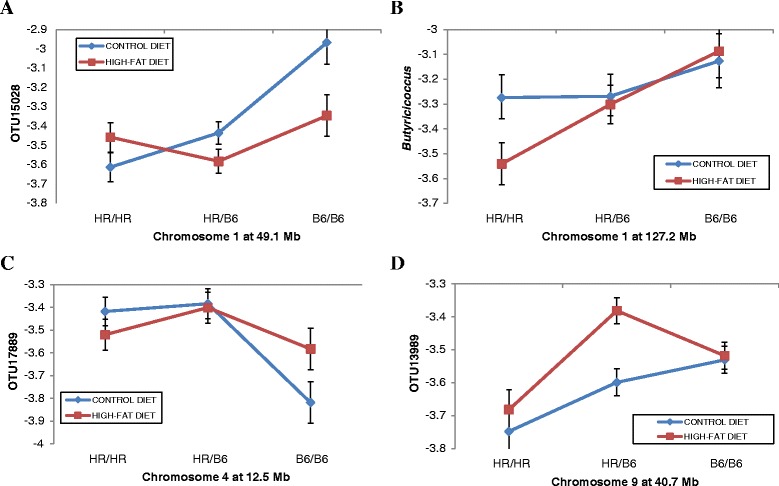
**QTL on Chr 1, Chr 4, and Chr 9 show complex patterns of gene X diet interactions. (A-D)** Plots of genotypic values at the QTL peak (X axis) are shown for four different OTUs where the QTL shows differential effects depending on whether the G_10_ mice were fed the control or the high-fat diet. Relative abundances of the OTUs for animals having the HR/HR parental, HR/B6 heterozygote, or B6/B6 parental genotypes at QTL peak are plotted on the Y-axis. **(A)** QTL for OTU15028 at 49.1 Mb on Chr 1. **(B)** QTL for Butyricoccus at 127 Mb on Chr 1. **(C)** QTL for OTU17889 at 12.5 Mb on Chr 4. **(D)** QTL for OTU13989 at 40.7 Mb on Chr 9. Error bars based on standard error.

A second set of overlapping QTLs showing significant interactions with diet are found on Chr 1 (Figure 5). Figure 5A shows that a Chr 1 QTL at 49.1 Mb clearly has a greater effect on the relative abundance of OTU15028 in mice fed the control rather than the high-fat diet whereas the reverse is true for the effects of a different QTL on Chr 1 (127.2) on *Butyricicoccus* (Figure 5B). Two additional examples are illustrated of QTLs with greater effects in mice fed the control (Figure 5C) or the high-fat diet (Figure 5D). The HR/HR genotype for the Chr 4 QTL (Figure 5C) increases the abundance of OTU17889 in mice fed the control diet, but the reverse is true for the B6/B6 genotype. The Chr 9 QTL affecting OTU13989 (Figure 5D) showed a different pattern in which the HR/B6 genotype increases the abundance of this taxon more so in mice fed the high-fat rather than the control diet.

### QTL analysis of IghV utilization patterns

The availability of ileal tissue from a large subset of the animals across both diets provided a unique opportunity to examine the role of expressed IgA on microbiota composition and to determine if host genetic influence on IgA rearrangements or their expression played any role in shaping microbiota composition. The abundance of transcripts from rearranged and expressed IgA receptors among B-cells resident in the ileal tissue were measured by pyrosequencing of amplicons generated from cDNA using a primer immediately upstream of the IgA constant region (IgAC) in combination with the universal variable region (Vh) primer. The resulting sequences were then binned initially by BLAST analysis of each read against the VH region repertoire and each bin was subsequently normalized by the total number of reads per animal. The means of the log-transformed abundances of these 67 IgA traits (Additional file 6) varied from -3.38 (B196, IghV3-4) to -1.226 (B59, IghV1-53) and accounted for >90% of the total reads across most mice. Over all individuals, however, the minimum value for the IgA abundances was -5.082 (B218, IghV5-6-2), and the maximum individual value was 0.015 (B79, IghV1-72). ANOVA showed no significant effect of diet on the IgA abundance.

Estimates of the variance components (Additional file 7) differ considerably among the 67 IgA expression traits, with values for the cohorts varying from 0 to 19.1% and being the least important (mean = 2.8%) of the four components. Parity (differences between successive litters) was slightly more important (mean = 3.6%), but these two sources of environmental variation (cohorts and parity) jointly contribute just 6.4% of the total variation. Family differences vary from 0 to 32.4% and average 12.1%, greater than that for the microbiome traits. Again, however, the largest contribution is from residual (within family) variation, the estimates in the range of 59.7% to 99.4%, and averaging 81.6%.

As shown in Table 2, QTL analysis of IghV utilization patterns identified a total of 56 QTLs that had LOD scores reaching at least the 10% experimentwise threshold. Only one QTL (affecting B81, IghV1-75) showed a marginally significant interaction with sex, and none significantly interacted with diet, which was expected given the lack of dietary effects on the individual IghV abundance. Remarkably, 36 of the 56 QTLs had LOD scores >10, with 34 of these highly significant QTLs localized to a segment on Chr 12 that encompasses the IgH region. In addition, two highly significant QTLs mapped to segments on Chr 17. While this 7 Mb confidence interval spans >400 genes/pseudogenes, it includes the mouse Major Histocompatibility locus (MHC) with 74 class I, II, and III genes. Given the known involvement of MHC in controlling immunoglobulin production, it seems reasonable that diversity in one of the class II genes could give rise to variation in the IgH V-region utilization patterns. With highly significant QTLs overlapping well-known sites contributing to regulation of immunoglobulin production, the IgA-specific IghV region utilization patterns appear to be robust phenotypes. However, none of the 56 overall QTL for IgA overlapped with any of the QTL for microbiota. Also, correlations between the abundances of each of these 67 IgA variable regions and the microbiota comprising the CMM, all were non-significant (*P* >0.05). Importantly, the lack of QTLs with pleiotropic effects on IgA and the microbiota implies that genetic variation influencing IghV region utilization and expression has little effect on broad compositional features of the microbiota in the mouse population that was studied.

**Table 2:** **QTL statistics for the IgA traits in the G**_**10**_ **mouse population**

**Trait**	***IghV*** **gene**	**Ch**	**Location (Mb)**	**CI (Mb)**	**LOD**	***A***	***d***	**%**	**Interaction**
B182	*IghV*2-6-1	1	70.1	69.2-73.1	4.21	**0.206**	**0.166**	6.44	
B48	*IghV*14-4	4	117.5	106.0-124.5	4.64*	0.037	**-0.234**	6.66	
		12	112.3	108.8-118.1	4.63*	**0.210**	-0.082	8.60	
B30	*IghV*1-26	6	137.8	130.6-143.3	4.59*	**0.221**	0.112	8.91	
		12	114.5	109.1-120.0	3.94	**0.147**	-0.022	7.47	
B122	*IghV*1S17	7	4.3	3.6-16.2	4.57*	**-0.139**	**0.188**	7.22	
B81	*IghV*1-75	8	91.5	90.6-92.8	4.07	**0.144**	**0.367**	6.51	Sex (M,**F**)
		12	114.5	114.0-120.0	21.29*	**-0.530**	**0.257**	38.01	
		19	42.8	42.1-47.7	3.94	**-0.197**	**0.199**	5.67	
B207	*IghV*5-12-1	9	27.3	18.8-32.9	3.98	-0.081	**-0.325**	6.37	
		12	114.5	114.0-120.0	26.11*	**0.689**	**0.306**	42.76	
B218	*IghV*5-6-2	9	22.8	13.7-31.8	4.13	-0.067	**-0.351**	6.65	
		12	114.5	114.0-120.0	24.85*	**0.701**	**0.271**	41.87	
B44	*IghV*14-2	12	114.5	114.0-120.0	13.74*	**-0.325**	**0.128**	23.17	
B76	*IghV*1-7	12	114.5	114.0-120.0	5.07*	**0.158**	-0.036	9.55	
B88	*IghV*1-81	12	114.5	114.0-120.0	19.31*	**-0.472**	**0.174**	36.27	
B89	*IghV*1-82	12	114.5	110.9-120.0	5.07*	**0.160**	0.019	10.26	
B57	*IghV*1-52	12	114.5	114.0-120.0	4.78*	**0.187**	-0.027	9.63	
B23	*IghV*1-22	12	114.5	114.0-120.0	10.35*	**-0.028**	**0.009**	20.36	
B17	*IghV*1-20	12	114.5	114.0-120.0	13.74*	**0.344**	0.030	26.70	
B37	*IghV*1-34	12	114.5	114.0-120.0	26.32*	**0.540**	**0.258**	43.19	
B14	*IghV*1-18	12	114.5	114.0-120.0	14.55*	**-0.337**	**0.141**	28.03	
B80	*IghV*1-74	12	112.3	108.8-118.1	6.12*	**-0.256**	0.058	11.84	
B42	*IghV*1-4	12	114.5	114.0-120.0	15.14*	**-0.432**	**0.142**	27.61	
B71	*IghV*1-63	12	114.5	114.0-120.0	7.87*	**0.300**	0.069	14.67	
B91	*IghV*1-84	12	114.5	114.0-120.0	23.21*	**0.505**	**0.170**	40.59	
B72	*IghV*1-64	12	114.5	114.0-120.0	42.12*	**-0.728**	**0.446**	55.90	
B54	*IghV*1-5	12	114.5	114.0-120.0	12.00*	**0.361**	0.061	23.32	
		13	37.6	35.4-41.8	4.50*	**-0.213**	0.031	8.41	
B87	*IghV*1-80	12	114.5	114.0-120.0	34.33*	**-0.700**	**0.488**	49.98	
B15	*IghV*1-19	12	114.5	114.0-120.0	8.66*	**-0.294**	**0.171**	16.25	
B78	*IghV*1-71	12	114.5	114.0-120.0	5.45*	**-0.279**	0.083	9.80	
B93	*IghV*1-9	12	114.5	114.0-120.0	52.78*	**-0.913**	**0.630**	70.88	
B61	*IghV*1-55	12	114.5	114.0-120.0	43.09*	**-0.791**	**0.484**	61.04	
B79	*IghV*1-72	12	114.5	114.0-120.0	46.20*	**-0.846**	**0.558**	61.53	
B73	*IghV*1-66	12	114.5	114.0-120.0	12.00*	**-0.382**	0.108	22.15	
B43	*IghV*14-1	12	114.5	114.0-120.0	9.53*	**-0.386**	**0.194**	16.72	
B55	*IghV*1-50	12	114.5	118.3-120.9	13.31*	**-0.436**	**0.166**	22.83	
B46	*IghV*14-3	12	114.5	114.0-120.0	52.85*	**0.979**	**0.529**	65.56	
		18	82.1	77.7-84.3	4.48*	**-0.421**	**0.675**	11.98	
B92	*IghV*1-85	12	114.5	114.0-120.0	31.70*	**-0.794**	**0.465**	51.35	
B64	*IghV*1-59	12	114.5	114.0-120.0	15.30*	**-0.541**	**0.172**	26.78	
B60	*IghV*1-54	12	114.5	114.0-120.0	27.34*	**-0.699**	**0.537**	45.98	
B82	*IghV*1-76	12	114.5	114.0-120.0	29.75*	**-0.606**	**0.338**	48.99	
B10	*IghV*1-14	12	114.5	114.0-120.0	29.75*	**1.098**	**0.843**	69.66	
B40	*IghV*1-37	12	114.5	110.9-120.0	4.02	**0.230**	-0.048	7.37	
B153	*IghV*1S61	12	114.5	114.0-120.0	62.17*	**1.189**	**0.733**	74.98	
B234	*IghV*6-3	12	114.5	114.0-120.0	5.60*	**-0.235**	**0.154**	11.23	
B83	*IghV*1-77	12	114.5	114.0-120.0	15.01*	**-0.400**	**0.254**	28.59	
B114	*IghV*1S135	12	114.5	114.0-120.0	43.97*	**0.988**	**0.471**	64.30	
B217	*IghV*5-6-1	12	114.5	114.0-120.0	34.60*	**-0.766**	**0.438**	54.28	
B223	*IghV*5-9	12	114.5	114.0-120.0	19.14*	**0.544**	**0.280**	33.41	
B63	*IghV*1-58	12	114.5	114.0-120.0	22.02*	**-0.667**	**0.222**	38.12	
B189	*IghV*2-6-8	12	114.5	114.0-120.0	9.23*	**0.376**	0.098	16.43	
B175	*IghV*2-3	12	114.5	114.0-120.0	14.48*	**-0.481**	**0.277**	26.82	
B196	*IghV*3-4	12	114.5	112.3-120.0	12.45*	**0.445**	0.100	24.31	
B75	*IghV*1-69	17	33.7	31.6-38.0	4.70*	**0.177**	0.108	8.02	
B195	*IghV*3-3	17	33.7	31.9-42.6	12.83*	**0.348**	**0.394**	18.80	
		17	45.6	43.6-48.7	12.81*	**0.447**	**0.495**	29.33	
		18	82.1	79.6-84.3	6.38*	**-0.445**	**0.724**	18.79	
B178	*IghV*2-4-1	18	53.9	52.9-58.5	4.25*	**0.089**	**0.173**	6.98	
B59	*IghV*1-53	19	42.1	37.9-42.8	4.20	-0.008	**-0.118**	5.73	

Shown are all QTLs affecting the IgA traits that had LOD scores reaching the suggestive or significant (*) experiment-wise level of significance. The chromosome (Ch) location of the peak and confidence intervals (CI) of each QTL is given in Mb (from NCBI Build 37). Also shown is the percentage contribution (%) of each QTL to the total variance of each trait, and its additive (*a*), dominance (*d*) genotypic effects (bolded values indicate significance). Sex (M,**F**) = an interaction of the QTL with sex, where the QTL is significant in both sexes but has a greater effect in females.

**Figure 6:**
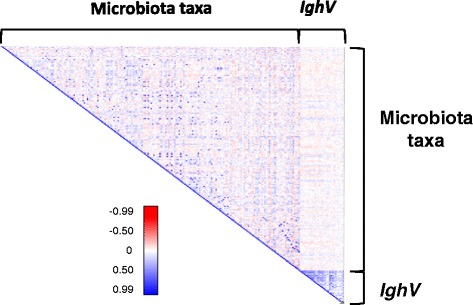
**Correlation matrix between microbiota composition and IghV region expression.** For each of 308 mice, the relative abundance of 67 IghV regions shared among the expressed IgA population of >75% of the animals and the relative abundance of the most abundant taxon of the microbiota were used to calculate the correlation coefficient (r). Red and blue coloring corresponds to the *r* value for each pair-wise comparison according to the color gradient.

Because antibody specificity is generated through recombination of the V, D, and J regions, along with hypermutation, it was possible that IghV region utilization alone did not provide the specificity necessary to detect association with the microbiota. To test this possibility, we employed a higher resolution approach to bin the IgA sequences, using the K-mer based strategy in CD-hit [39] to cluster predicted protein sequences from the 2,644,330 quality-filtered IgA reads. With a 99% identity cutoff for clustering, this yielded 4,505 different clusters. The vast majority of these clusters exhibited low abundances and were often present in only a single animal, or were sparsely distributed. However, 71 clusters were observed across the majority of the animals (>5 reads shared across 75% of the mice), presumably arising from convergent clones expanded across multiple animals. Some correlations of the abundances of these 71 clusters with the 300 most abundant species and OTUs from the 16S rRNA-derived microbiota data (Figure 6) were significant (*r* >0.5) among several of the IgA clusters themselves and among several of the individual microbial taxa, but no significant associations were observed between any of the IgA clusters and microbial taxa. The lack of overlapping QTLs and the absence of correlation collectively imply that genetic variation influencing the immunoglobulin repertoire plays little role in the individuality of microbiome composition.

## Discussion

Our study population presented a unique opportunity to examine a combination of deterministic factors that shape composition of the gut microbiome in G_10_ descendants of an advanced intercross population that had previously been studied at G_4_. Several aspects of the overall microbiome composition were notably different between the G_4_ and G_10_ animals. While the overall species compositions differed significantly (substantially higher in members of the *Bacteriodetes* at G_10_ versus G_4_), the most striking difference was the variation between breeding cohorts, accounting for an average 26% of the total variation across taxa in the G_4_ but only 9.6% of the total variation in the G_10_. It is possible that changes in the pyrosequencing reagent stream that were introduced by the supplier during the 18 months between the G_4_ and G_10_ populations contributed to the unique compositional features, but these changes would most likely manifest as biases in taxonomic compositions and not their distributions across the populations. Resequencing a small number of G_4_ samples with similar reagents used for G_10_ samples showed quite similar taxonomic content, suggesting this was not a factor. Second, it is possible that population-specific characteristics of the microbiota were brought about by phenotypic and/or genotypic drift or they reflect the degree to which recombination has dispersed the variation from parental lines across the progeny. For the latter case, the dispersal of parental genomic variation through accumulating recombinations by G_10_ could result in a more evenly distributed microbiota. The increased dispersion of genomic variation could also be augmented independently by ‘maturation’ of the microbiome, going from more chaotic distributions during the first few generations in the facility to more stable configurations after 10 generations of breeding in the same facility.

### Effect of high-fat diet on the gut microbiota of the G_10_ population

A high-fat diet was incorporated into the experimental design to test for interactions between genotype and diet. This design also provided an opportunity to examine closely effects of the high-fat diet alone across an intercross population, in contrast to studies using a single inbred line. Single line studies often show substantial changes in the microbiota [31] marked by blooms of related taxa, whereas the effects of a high-fat diet across the large numbers of animals from our intercross population showed a modest effect on alpha-diversity and small, but statistically significant differences across a large number of taxa. Whether the magnitude of the diet effect was muted in our study because of the genetic diversity from the intercross or some other factor is not clear. Recent studies across 100 different mouse lines showed dietary effects dispersed across several taxa and these effects were unique to certain lines [40]. Clearly, understanding the effects of diet on the microbiome will require much more study in different types of populations to understand these complex interactions.

### Microbiota QTLs

The results of our analysis defined 42 QTLs that affected the relative abundances of 39 of the 203 taxa. We were conservative in using only 5% and 10% genome-wide thresholds rather than chromosome-wide thresholds to determine significance. Because we analyzed so many traits, however, it was not surprising that the FDR procedure suggested that as many as roughly 1/2 of the QTLs affecting these traits may be false positives. On the other hand, this also means that about 20 QTLs reflect true underlying genetic variation affecting the microbiota composition. The greatest support (lowest FDR values) was for QTLs on Chr 9, especially one at 37.3 Mb affecting OTU41353.

The mapping precision of the QTLs we discovered was enhanced in our advanced intercross population at G_10_ compared to that in the G_4_ population. Thus the mean confidence interval of 9.85 Mb calculated for these QTLs is considerably less than that of 20.7 Mb found by Benson *et al.* [10] in the G_4_ mice, and would have dropped to about 7 Mb if we had used a 1-LOD (rather than 1.5-LOD) drop criterion as was done in the G_4_ study. It therefore is clear that the additional mapping precision expected from the accumulated recombinations in the G_10_ population was in fact achieved.

### QTL replication

Despite the population-specific features of the G_4_ and G_10_ microbiota, normalizing the levels of taxonomic inquiry in the G_4_ and G_10_ microbiotas produced four different genomic segments where QTLs overlapped from the two studies. At three of these loci, the taxa controlled in the G_4_ or G_10_ populations shared taxonomic relatedness at the family or order level. In addition to the replication we observed in these populations, recent QTL analyses of the skin microbiome identified two out of the 14 QTLs controlling taxa of the skin microbiome that overlap those we had previously found in the G_4_ population [41]. Of the two shared QTLs, only the QTLs on Chr 14 appear to control taxonomically related organisms (G_4_ QTL controls *Lactococcus* whereas the skin microbiome QTL controls an OTU belonging to the *Firmicutes*).

From a broad perspective, the ability of host loci to control a variety of microbial taxa would support multiple possible outcomes of microbiome assembly, with each assemblage potentially sharing a common core of metabolic and functional niches despite the diversity in taxonomic composition. From the host perspective, the ability to support multiple possible assemblages would be advantageous and allow the assembly process to work upon the microbial capital it happens to encounter early in life.

### Pleiotropic patterns

A prominent feature of several QTLs we discovered was that they affected more than one taxon. While it is possible that some of this apparent pleiotropy is due to linkage disequilibrium, it seems unlikely that this would explain all of the pleiotropic loci. Correlated traits are often related by their contribution to similar pathways or functions, but in the case of microbial traits, correlated microbial taxa could be controlled by the same QTL due to common physiological characteristics (for example, common sensitivity to defensins secreted in the mucosa), or common metabolic traits (for example, ability to degrade mucins). One could even envision pleiotropy occurring indirectly, whereby host genetic factors favor colonization by a given taxon and this initial event sets the stage for colonization by a second taxon (for example, metabolic end product of one taxon serving as a substrate for a second taxon). This could be the case at the complex of overlapping QTLs we identified on Chroe 9. Here we observed distinct effects on two different sets of correlated taxa (Figure 3). While both sets of correlated taxa (colored red or blue in the matrix) comprise OTUs belonging to the class *Bacteriodetes*, early colonization by OTU17740 (blue cluster), may favor subsequent colonization by OTUs 25269 and 25483 whereas colonization by OTU41353 favors subsequent colonization by 29084. Colonization by *Peptococcus* (OTU13989) may actually favor a third pathway in which strains belonging to the red or blue correlated clusters are tolerated. Defining the underlying basis of these QTLs will therefore provide clues to important characteristics of gut microbes and the niches that they occupy.

### Microbiota QTLs, obesity, and diet

Given the known association of gut microbes with obesity and various metabolic disorders [42], it is reasonable to expect that some of the microbiota QTLs might exhibit pleiotropic effects on body weight or composition. To examine this possibility, we compared the locations of the microbiota QTLs (Table 1) with QTLs previously found for body weight and the percentage of fat and lean tissue in these same (8-week-old) mice [33]. This comparison revealed four instances of overlaps for QTLs on Chr 5, Chr 9, Chr 11, and Chr 18, details of which are summarized in Table 3. Several potential candidate genes for these QTLs are listed in the Table, but it will require additional effort to discover whether these or other genes underlying the QTLs actually affect both kinds of traits, and if so, what pathways might be involved.

**Table 3:** **Possible candidate genes for QTLs affecting microbiome and body weight/composition traits in the G**_**10**_ **mice**

**Chrom**	**Microbiome trait**	**Position (Mb)**	**CI (Mb)**	**Body trait**	**Position (Mb)**	**Candidate gene(s)**	**Position (Mb)**
5	OTU3615	115.2	112.9-118.1	% Fat	116.6	*Pla2g1b*	115.4
						*Nos1*	117.8-117.9
9	*Lactococcus lactis*	113.3	112.3-115.0	Weight	112.3	*Glb1*	114.4
11	OTU22207	97.8	93.4-114.0	% Lean and % Fat	105.1 and 107.3	*Igf2bp1*	96.0
						*Gast*	100.3
						*Pyy*	102.1
18	OTU30840	68.4	65.4-70.2	Weight, % Lean and % Fat	69.1 and 69.6	*Mc4r*	66.9

For each of four chromosomes (Chrom), the positions in Mb are shown for QTLs affecting microbiome abundances and body weight and composition traits as well as possible candidate genes for these QTLs. The confidence intervals (CI) also are given for the QTLs affecting the microbiome traits.

Regardless of which candidate genes contribute to these phenotypes, our discovery of putative pleiotropic effects of QTLs on microbiome composition and body weight/fatness/leanness illustrates the theoretical potential for genetic predisposition to obesity to be manifested in part by susceptibility to aberrant colonization of the gut.

Perhaps the most significant finding in our study was the identification of several microbiota QTLs exhibiting interactions with diet. While only eight of the 42 total microbiota QTLs (19%) showed these interactions, this low proportion is identical to that for QTLs affecting body weight or the percentage of fat or lean tissue in this same mouse population [32]. Because of the apparent pleiotropy of these QTLs, however, as few as four different genes (two on Chr 1, one on Chr 4, and one on Chr 9) may be involved.

Among the microbiota QTLs showing interactions with the dietary environment, the four on Chr 9 each affecting a different taxon were most impressive. These QTLs all mapped to the same precise position (40.7 Mb), and thus likely represent the same underlying gene. The QTL affecting OTU13989 showed the most restricted confidence interval of just 1.9 Mb that according to the Mouse Genome Informatics database [43] contains 11 protein coding genes. Of these 11, *Bsx*, *brain specific homeobox* (at 40.9 Mb), would seem to represent an outstanding candidate for the QTLs. *Bsx* mutants exhibit increased fat mass, decreased food intake after fasting, and reduced locomotor activity [44].

From a broader perspective, our discovery of gene X diet interactions on microbiota composition supports the idea that dietary modifications can potentially modify or even overcome allelic effects on microbiome composition. In fact, recent studies on the microbiome of infants show that dietary modulation of microbiome composition and function can influence expression patterns of innate response genes [45]; and in adults, dietary modulation can also affect metabolic and inflammatory markers in the blood [35]. Combined, these findings are of special significance to human health because they suggest that dietary intervention could overcome heritable components of disease predisposition that are manifest through the gut microbiome. Similarly, with respect to animal agriculture, our discovery implies that dietary modulation could overcome the effects of undesirable genotypes associated with weight gain or even with colonization by zoonotic pathogens.

### The microbiota and IgA

Secretory IgA (SIgA) plays important roles in barrier defense against enteric pathogens by binding to cell surface molecules of the pathogen and precluding attachment [46,47]. Such a barrier defense would not necessarily be limited to pathogens and could play a role in homeostasis by limiting exposure of the epithelial layer to the mass of microbial cells in the microbiota. Indeed null mutations that block class switching to IgA have significant effects on microbiota composition [48,49]. More recently, FoxP3+ Tcell-dependent production of high-affinity IgA was found to be associated with shaping the microbiota, specifically by enriching for members of the *Firmicutes* [29]. Remarkably, this IgA-mediated enrichment seems to be mediated through a positive influence of the IgA on the microbiota as opposed to the removal of potential competitors.

Unlike studies in isogenic derivatives of a single line, our study provided a unique opportunity to examine specificity of the expressed IgA repertoire with respect to the microbiome across a population with genetic diversity dispersed randomly across the progeny. Genetic variation had a significant outcome on variable region utilization patterns but it did not affect composition of the gut microbiome. Likewise, we could not detect association between VDJ rearrangements and composition of the contemporary microbiota. Of course, it is quite possible that specificity of IgA-microbe interaction is below our level of sensitivity. While we can approximate species-level resolution with our OTU-pipeline, specificity of the interaction may be dictated at the strain-level. The IgA-mediated enrichment of the microbiota observed by Kawamoto *et al.* [29] was detected by sequencing of antibody-bound taxa, implying that the high-affinity IgA responsible for shaping the microbiota in their studies was directed toward cell surface molecules. Indeed, cell surface molecules such as teichoic acids, extracellular polysaccharides, and surface proteins tend to be some of the most highly variable and strain-specific traits of a bacterial species, making it unlikely that we would have detected such interactions. In the absence of strong associations between the microbiome composition and expressed IgA molecules, the correlation among Vh usage patterns and convergent VDJ rearrangements that we observed across individuals becomes even more intriguing. Convergence among expressed VDJ regions between individuals has been observed in antibody repertoires of zebrafish [50] and mice [51] and it can be observed in vaccine responses as well as anamnestic sera from patients recovering from epidemics, implying that microbes may be capable of eliciting specific signatures of IgH rearrangements. If so, then the convergent responses observed in our animal population could either reflect signatures of strain-level interactions between the contemporary microbiota and the mucosal immune system or, they could reflect interactions with the microbiota early in life, prior to contemporary microbiota we measured in the mature animals.

## Conclusions

Detailed analysis of the taxonomic abundance of the gut microbiota at G_4_ and G_10_ of the C57BL/6J X HR intercross have provided insight into the impact of host factors, dietary factors, and stochastic factors on gut microbiota composition. Major differences in dominant taxa of the gut microbiota occurred over time between G_4_ and G_10._ This was particularly the case for the distributions of these taxa, which were highly cohort-dependent and variable (wide ranges) in G_4_ animals but less cohort-driven with modest ranges at G_10_, suggesting that the microbiome may have progressed from a more to less chaotic assembly over time. Despite these differences, four overlapping QTLs were still detected among both G_4_ and G_10_ mice.

A high-fat diet in one-half of the G_10_ animals brought about a modest impact on the microbiota that resulted from cumulative incremental changes in many taxa as opposed to large swings in taxonomic abundance. The genomic region at 40.7 Mb on Chr 9 had overlapping G_4_/G_10_ QTLs and many of the G_10_ QTLs in this region showed significant interactions with diet, as did additional QTLs on Chr 1 and Chr 4. Detection of these gene X diet interactions implies that it may be possible to modify the heritability of microbiota composition via dietary modulation.

Quantitative analysis of the patterns of Vh utilization in the expressed IgA transcripts of G_10_ animals showed a remarkable number of convergent VDJ rearrangements that were shared between individuals. The convergence could reflect common exposure of earlier assemblages of the microbiota as no associations were detected among the Vh utilization patterns and any of the microbiota that were measured contemporary with the Vh patterns. On the other hand, very high degrees of association were detected in the Vh utilization patterns and genetic variation in regions of Chr 12 and Chr 17 that overlap with the IgH and MHC loci. Although genetic variation in these major drivers of immunoglobulin responses had expected effects on variation in VDJ rearrangements, none of this variation accounted for variation in the contemporary microbiota and correspondingly, no overlapping microbiota/Vh were detected. Collectively, we conclude that host genetics and diet converge to shape microbiota composition, but the effects of host genetic variation are not manifest through Vh utilization patterns for immunoglobulin A.

## Materials and methods

### The population

The population of mice used in this study was generated from original crosses of inbred C57BL/6J (B6) female mice with male mice from a strain (HR) selected for a high level of voluntary wheel running [52]. The mice were reared through the ninth generation following a previously-described protocol [53], at which time single-pair matings were made that produced up to two litters each in the G_10_ generation. All G_10_ pups were weaned at 3 weeks and by 4 weeks, randomly allocated into either a group fed a high-fat diet or a group fed a control diet (see Table 1 in [53]). When the mice were approximately 8 weeks of age, fecal pellets were collected for DNA extraction and subsequent pyrosequencing. Mice then were given access to running wheels during each of 6 consecutive days, with exercise traits measured for all individuals in one of 13 different sequential cohorts as previously described [53]. All G_10_ mice were sacrificed shortly after the exercise period (between age 53 to 59 days), tail clips were taken for genotyping and segments of the ileum were removed for RNA extraction (described below). All procedures were approved by the Institutional Animal Care and Use Committee at the University of North Carolina at Chapel Hill.

### SNP genotyping

We used the Mouse Universal Genotyping Array, MUGA [54], to yield genotypes for 2,058 fully informative SNPs (average spacing = 1,223 kb). SNPs were checked for significant segregation distortion, and for errors using Merlin [55], with extremely unlikely calls dropped from the analysis. A list of these SNP markers with their locations (in Mb) is given in an Appendix in Leamy *et al.* [53]. Genotypes of the individual animals are available at the CAGE microbiome analysis database [56].

### Pyrosequencing of microbiota

DNA extraction from fecal pellets and pyrosequencing analyses were performed as previously described [10,57]. Composition of the microbiota was assessed by deep pyrosequencing of PCR products originating from the V1-V2 region of the 16S rRNA gene with bar-coded fusion primers containing Roche-454 A or B Titanium sequencing, followed by a unique 8-base barcode sequence (B) and, finally, the 5′ ends of primer A-8FM (5′-CCATCTCATCCCTGCGTGTCTCCGACTCAGBBBBBBBBAGAGTTTGATCMTGGCTCAG) and of primer B-357R (5′-CCTATCCCCTGTGTGCCTT-GGCAGTCTCAGBBBBBBBBCTGCTGCCTYCCGTA-3′). All PCR reactions were quality controlled for amplicon saturation by quantifying and comparing band intensities of the PCR products after gel electrophoresis with standards using GeneTools software (Syngene). Amplicons from 48 individual samples were pooled in equal amounts, gel-purified, quantified by Pico Green analysis, and used for emulsion PCR (emPCR). After recovery and enrichment for DNA-containing beads, the emPCR products from the 48-sample pools were sequenced on individual regions of 2-region Picotitre plates on a Roche-GS-FLX machine using Titanium sequencing chemistry.

### Pyrosequencing data processing pipelines

Raw data from the Roche-454 GS-FLX machine were first processed through specialized scripts that filtered the data on the basis of the following criteria, with sequences not meeting these criteria being removed from further analysis: (1) a complete forward primer sequence and barcode; (2) ≤2 ‘N’ in a sequence read, where N is equivalent to an interrupted and resumed sequencing signal from sequential flows; (3) a sequence of >200 NT and <500 NT; and (4) an average quality score ≥20 across the entire length of the sequence.

After filtering, reads were trimmed to remove 5′ and 3′ adapter and primer sequences, parsed by barcode into corresponding sample files, automatically associated with a matching .QUAL file containing the quality scores, and uploaded into a MySQL database and associated with sample information. MySQL database tables are stored on a database server and available to the public through the CAGE microbiome analysis database login [58]. The raw read and .QUAL files are also available at the NCBI Sequence Read Archive under Bioproject Accession PRJNA265870. To help normalize taxonomic assignment and phylogenetic distance estimates of individual sequence reads, the entire data set was initially processed through the Multi-CLASSIFIER algorithm, which assigns hierarchical taxonomic status to each sequence read based on a covariance model developed from a training set [59,60]. This algorithm is capable of processing very large data sets and was recently shown to provide adequate taxonomic assignments to pyrosequencing data [61]. After processing through the Multi-CLASSIFIER, sequences were parsed into ‘classified’ and ‘unclassified’ sets based on meeting threshold limits of 0.8 at the genus level against the Multi-CLASSIFIER model.

Classified reads were then assigned species-level status using a BLAST pipeline that associated the read with species-level taxonomic assignment using a curated database developed from RDP and SILVA databases of curated 16S ribosomal RNA sequences [59,62]. Sequences were considered a species match if they achieved 97% identity with a reference sequence over a minimum of 200 bases of contiguous BLAST alignment. Sequence reads that failed to meet the 0.8 scoring threshold at the genus level from the Multi-CLASSIFIER algorithm (‘unclassified’ reads) were further processed into Operational Taxonomic Units (OTUs) using CD-Hit to estimate phylogenetic distances and cluster at 97% cutoff [63]. Taxonomic status of these OTUs was approximated by BLAST against the curated database. For QTL mapping, only dominant taxonomic/OTU bins containing at least five sequences in >75% of the mice were used. This reduced the total number of taxonomic/OTU bins from >18,000 to 203 bins that were log-normally distributed and referred to herein as the Core Measurable Microbiota (CMM). In addition to removing sparse data, this threshold step also had the important function of removing bins that result from chimeric sequences, artifacts of aggressive clustering, or sequencing errors. Reads from each bin from the combined ‘classified’ and ‘unclassified’ portions of the pipeline were then normalized relative to the total number of reads for each sample. For mapping and statistical analyses, the abundances were subjected to log_10_ transformation to reduce the effects of extensive variation in values across multiple mice. Microbiota data were available for a total of 472 mice. Raw data are available at the database server [58] and at the NCBI Sequence read Archive under Bioproject Accession PRJNA265870.

### Pyrosequencing of expressed IgA transcripts

RNA was extracted from flash-frozen segments of the ileum using the Biosprint One-for-all Vet Kits (Qiagen). Ileum segments were suspended in 1 mL of Trizol in 2 mL Cryovials along with a single 3 mm sterile tungsten carbide bead (Qiagen). Samples were homogenized for 4 min at 30 cycles/s in a Tissue Lyzer and immediately placed on ice. After a 3-min centrifugation at 14,000 rpm, 300 uL of the supernatant was transferred to individual wells of the One-for-all Vet kit 96 deep well plates and the remainder was archived at -80°C. The deep well plates were then loaded onto the Biosprint 96 and automated RNA extraction performed according to the manufacturer’s instructions and purified RNA was eluted into RNAse-free water. After quantification, cDNA was prepared from 5 ug of total RNA using oligo-dT(12-18) primers (Invitrogen) and the Superscript III protocol (Invitrogen). The resulting cDNA was diluted 1:10 into 50 uL PCR reactions containing 10% DMSO along with 0. 6 μM of PCR primers for the IgA constant region (IgAC) [64] and a universal primer for the Igh variable region (Universal Vh) [65]. The IgAC and Universal Vh primers also contained the Roche A and B Titanium adapter sequences (bold) at their 5′ ends. Primer sequence for the Roche B adapter- IgAC primer is **CCTATCCCCTGTGTGCCTTGGCAGTCTCAG**CTCAGGCCATTCAGAGTACA. The primer sequences for the Roche A-universal Vh primers also contained a sample-specific 8-base barcode (b) immediately upstream of the Vh region. The primer sequences for the Roche A-barcode-Universal Vh primers were: **CCATCTCATCCCTGCGTGTCTCCGACTCA**GbbbbbbbbAGGTSMARCTGCAGSAGTCWGG. PCR amplification was performed in 20 mM Tris-HCl (pH 8.4), 50 mM KCl, 1.5 mM MgCl2, 2.5 U TaqDNA polymerase (Invitrogen Life Technologies), and 0.2 mM each of dGTP, dATP, dTTP, and dCTP. The PCR amplification program consisted of 30 cycles of 30 s at 94°C (2 min in first cycle), 1 min at 58°C, and 1 min at 72°C. The program was followed by 10 min at 72°C to allow extension of all products. After PCR amplification and quality control check by gel electrophoresis, the amplicons were quantified by Pico-Green and pooled at a 1:1 ratio in pools of 48 samples each followed by two cycles of cleanup using Ampure beads. Each pool was then subject to pyrosequencing on the Roche-454 FLX Titanium platform. Raw data are available at the database server [58] and at the NCBI Sequence read Archive under Bioproject Accession PRJNA265870.

To process the IgA sequence data for QTL analysis, the data were first filtered to remove low quality reads as for 16S rRNA sequencing. For each read, the predicted amino acid sequence of the appropriate reading frame was subsequently mapped by BLAST analysis against the 268 mouse Vh region genes from the ImMunoGene Tics web resource (IMGT) repertoire [66]. This yielded 67 IghV regions that were detected across 75% of the animals. For mapping, the relative abundance of transcripts from each IghV region bin for each animal was normalized by the total reads in each sample and log_10_-transformed.

### Preliminary statistical analysis

The log-transformed values for all microbiome and IgA traits first were subjected to a multivariate analysis of variance that showed overall significance (*P* <0.05) for sex, diet, cohort, parity, and litter size at birth. We therefore adjusted for the effects of these factors and examined the distributions of the abundances of the residuals for each trait. Using an alpha level of 0.01, and the false discovery rate [67] to adjust the probabilities from Kolmorgorov-Smirnov tests, all traits were found to be normally distributed. We therefore calculated means and standard deviations for all taxa to provide a basic description of their distributions.

It also was of interest to estimate variance components for families, parity, and cohorts to determine the contribution of each of these random factors to the total variance of each trait. Cohort and parity (differences between first and second litters in each family) effects are due to environmental and/or epigenetic factors whereas differences among families and within litters (residual) are produced by both genetic and environmental factors. We estimated cohort, family, parity, and residual components and tested them for significance via a mixed model that also included sex, diet, and litter size as fixed factors. Once calculated, we also expressed each of the four components as a percentage of the total variance.

### QTL mapping

G_4_ data were mapped as described [10] with R-QTL and adjusted for familial structure using the GRAIP algorithm to adjust the significance thresholds. To map QTLs in the G_10_ for the microbiota and IgA expression traits, we used the newly developed QTLRel program implemented in R [68,69] with an approach previously described [53]. This program was specifically developed to account for family structure and relatedness among individuals, as occurs in advanced intercross populations, and obviated the need for GRAIP-adjustments to the significance thresholds. We used the Haley-Knott interval mapping [70] option in QTLRel to impute genotypic values between any of the 2,058 total SNPs separated by more than 1 centiMorgan (cM), effectively increasing the total number of markers to 3,023. At each of these markers, QTLRel evaluated the phenotypic values of each trait with a model that included additive and dominance genetic effects as well as sex, diet, litter size, parity, and cohort to adjust for any effects of these covariates. The program produced likelihood ratio values at each of the markers throughout the genome that were converted into LOD scores.

To evaluate all of these LOD scores for each trait, we estimated both 5% (significant) and 10% (suggestive) genomewide thresholds with the traditional permutation method [71] available in QTLRel. For both the microbiota and the IgA expression traits, we ran the permutation procedure with 1,000 iterations on each taxon and recorded the 95th and 90th percentile LOD values in each of these runs. In the QTL scans for each trait, the highest LOD score on each Chr that met or exceeded the suggestive threshold was considered to represent the site of a putative QTL. Where the LOD score distributions exhibited multiple peaks exceeding this value, each peak was considered to represent the position of an individual QTL if it was separated by a drop of at least 1.5 LOD units from other peaks. Confidence intervals for each of the QTLs also were defined by 1.5 LOD drops on either side of the peak position [72].

Because we performed multiple (203) QTL scans, we expected a number of false positive QTL results by chance alone. To assess how probable this was for each of the putative QTLs found, therefore, we subjected the probabilities (estimated from permutations) associated with their LOD scores to the false discovery rate procedure [67]. We used an n = 203 in this procedure, and it yielded a false discovery rate (FDR) for each QTL that was useful in indicating its probability of being a false positive result.

QTLRel also computed additive (*a*) and dominance genotypic values (*d*) at the site of each QTL, and tested these values for significance (*P* <0.05) via individual *t*-tests. An additive genotypic value estimates one-half of the difference between the phenotypic values for the two homozygotes, which if positive in sign, indicates that the HR allele increases the mean of the trait (if negative, it decreases the mean). A dominance genotypic value estimates the difference between the mid-homozygous and the heterozygous values, and if significant, indicates that the QTL exhibits dominance [73]. To determine the extent and type of dominance, it is useful to divide *d* by *a*. Thus a *d*/*a* ratio of approximately +1 or -1 indicates complete dominance, a ratio well over +1 (>1.5) indicates overdominance (heterozygote greater than either homozygote), and a ratio well less than -1 (<-1.5) indicates underdominance (heterozygote less than either homozygote [74]. Besides *a* and *d* values, QTLRel also estimated the percentage of the total phenotypic variation of the trait explained by each QTL.

Once QTL locations were determined, we used an option in QTLRel to test for potential interactions of the QTLs with sex and with diet. At each of the sites of the QTLs discovered, QTLRel calculated the -2 ln (likelihood) for a model containing all terms described above, but in addition, the interactions of the *a* and *d* effects with sex (or diet). Each likelihood value generated from this model was compared with that generated in the null model that did not include the interaction terms, and the differences between these likelihoods were evaluated using a chi-square test. Probabilities from these tests were evaluated using the conventional level (0.05) of significance [53,74]. We interpreted significant QTL by sex (or diet) interactions as indicating different genotypic effects on the trait depending on the level of sex (males or females) or diet (control or high-fat). Where these interactions occurred, we tested the effect of the QTL in the separate sexes or diets and used the suggestive threshold values to assess significance.

### Data availability

Sequencing data and associated sample metadata are available at the NCBI archive under Bioproject Accession number PRJNA265870. Raw and processed sequencing data and metadata are also available at (http://gutmicro.unl.edu/ClientLogin/login.php). Complete instructions for using this database are available on the login page. Links to Excel files containing the processed microbiota data and processed genotype data are also available directly on the login page (http://gutmicro.unl.edu/ClientLogin/login.php).

## Additional files

Additional file 1:
**Table showing the basic statistics for the abundances of the 203 microbiota taxa.**
Additional file 2:
**Table showing the contributions of four variance components to the total variation in the abundances of the 203 microbiota taxa.**
Additional file 3:
**Figure illustrating a phylogenetic analysis of the microbiota taxa abundances in the G**_**4**_ **and G**_**10**_ **mouse intercross generations.**
Additional file 4:
**Table showing the basic statistics for the G4 taxa processed through the same OTU pipeline as the G10 taxa.**
Additional file 5:
**Table showing the QTL statistics for the G4 traits showing significant QTLs.**
Additional file 6:
**Table showing the basic statistics for the 67 IgA expression traits.**
Additional file 7:
**Table showing the contributions of four variance components to the total variation in the 67 IgA expression traits.**
