# Supplementary material for: Host genetics and diet, but not immunoglobulin A expression, converge to shape compositional features of the gut microbiome in an advanced intercross population of mice
Source: Genome Biol. 2014 Dec 17;15(12):552. doi: 10.1186/s13059-014-0552-6 (PMC4290092; doi:10.1186/s13059-014-0552-6)
Supplement: Additional file 1: — Table showing the basic statistics for the abundances of the 203 microbiota taxa. [file 13059_2014_552_MOESM1_ESM.pdf]

Additional file 1. Means, standard deviations (STD), and minimum (Min) and maximum (Max) values of the log of the of the proportion of sequence reads for each taxon. The sample size = 472 in all cases.

| Taxon | Mean | STD | Min | Max |
|---|---|---|---|---|
| *Alistipes* | -1.054 | 0.156 | -2.279 | -0.703 |
| *Bacteroides* | -1.232 | 0.250 | -2.274 | -0.614 |
| *Parabacteroides* | -1.358 | 0.266 | -2.533 | -0.584 |
| *Helicobacter* | -1.459 | 0.360 | -2.939 | -0.489 |
| *Oscillibacter* | -1.793 | 0.226 | -2.500 | -1.173 |
| *Odoribacter* | -2.082 | 0.228 | -3.106 | -1.587 |
| *Dorea* | -2.162 | 0.225 | -2.821 | -1.312 |
| *Lactococcus* | -2.264 | 0.345 | -3.848 | -1.397 |
| *Lactobacillus* | -2.295 | 0.641 | -4.149 | -0.566 |
| *Mucispirillum* | -2.679 | 0.582 | -4.692 | -0.956 |
| *TM7_genera_incertae_sedis* | -2.778 | 0.602 | -4.682 | -1.301 |
| *Ureaplasma* | -2.828 | 0.598 | -4.655 | -0.597 |
| *Roseburia* | -2.976 | 0.444 | -4.207 | -1.755 |
| *Butyricicoccus* | -3.298 | 0.504 | -4.618 | -2.085 |
| *Turicibacter* | -2.925 | 0.802 | -4.687 | -0.887 |
| *Rikenella* | -3.050 | 0.677 | -4.887 | -1.430 |
| *Alistipes_OTU15* | -1.347 | 0.176 | -2.697 | -0.915 |
| *Alistipes_OTU13* | -1.389 | 0.169 | -2.489 | -0.970 |
| *Bacteroides_OTU3* | -1.513 | 0.338 | -3.089 | -0.667 |
| *HelicobacterganmaniT* | -1.503 | 0.410 | -3.757 | -0.502 |
| *Oscillibacter_OTU3* | -1.869 | 0.232 | -2.653 | -1.198 |
| *Parabacteroides_OTU3* | -1.783 | 0.432 | -3.145 | -0.654 |
| *Parabacteroides_OTU6* | -2.084 | 0.664 | -4.165 | -1.021 |
| *Odoribacter_OTU6* | -2.260 | 0.229 | -3.209 | -1.722 |
| *Bacteroides_OTU12* | -2.048 | 0.578 | -4.219 | -0.819 |
| *Parabacteroides_OTU2* | -2.297 | 0.414 | -3.463 | -1.122 |
| *Dorea_OTU7* | -2.444 | 0.238 | -3.444 | -1.879 |
| *LactMG1363* | -2.336 | 0.361 | -3.968 | -1.428 |
| *Bacteroides_OTU13* | -2.428 | 0.419 | -3.874 | -1.020 |
| *Lactobacillusapodemit* | -2.502 | 0.625 | -4.185 | -0.613 |
| *Odoribacter_OTU1* | -2.859 | 0.272 | -3.974 | -2.219 |
| *Mucispirillumschaedlerit* | -2.679 | 0.582 | -4.692 | -0.956 |
| *Dorea_OTU12* | -2.726 | 0.447 | -4.155 | -1.318 |
| *Bacteroides_OTU0* | -2.860 | 0.426 | -4.340 | -1.855 |
| *Oscillibacter_OTU14* | -3.012 | 0.402 | -4.291 | -2.018 |
| *UreaplasmaurealyticumT* | -2.863 | 0.582 | -4.651 | -0.991 |
| *LactococcusSK11* | -3.236 | 0.404 | -4.383 | -2.383 |
| *Alistipes_OTU2* | -3.338 | 0.382 | -4.537 | -2.013 |
| *Helicobacter51449* | -3.152 | 0.554 | -4.472 | -1.494 |
| *Oscillibacter_OTU6* | -3.401 | 0.509 | -4.571 | -2.238 |

| | | | | |
|---|---|---|---|---|
| *Butyricicoccus_OTU7* | -3.320 | 0.511 | -4.631 | -2.104 |
| *Roseburia_OTU7* | -3.225 | 0.476 | -4.595 | -1.897 |
| *Lactobacillus33200* | -3.240 | 0.781 | -4.763 | -0.726 |
| *TM7_genera_incertae_sedis_OTU143* | -3.378 | 0.561 | -4.730 | -1.903 |
| *Parabacteroides_OTU8* | -3.589 | 0.380 | -4.462 | -2.651 |
| *Parabacteroides_OTU9* | -3.395 | 0.388 | -4.497 | -2.371 |
| *Odoribacter_OTU2* | -3.270 | 0.489 | -4.671 | -2.210 |
| *Bacteroides_OTU7* | -3.328 | 0.585 | -4.688 | -2.046 |
| OTU4825 | -1.505 | 0.324 | -4.232 | -0.875 |
| OTU12405 | -1.662 | 0.254 | -3.532 | -1.160 |
| OTU13263 | -2.166 | 0.318 | -4.289 | -1.532 |
| OTU10148 | -2.283 | 0.424 | -4.199 | -1.084 |
| OTU20373 | -2.385 | 0.288 | -3.627 | -1.600 |
| OTU26285 | -2.485 | 0.387 | -4.042 | -1.608 |
| OTU20360 | -2.476 | 0.414 | -4.210 | -1.633 |
| OTU27073 | -2.479 | 0.455 | -4.178 | -1.536 |
| OTU21224 | -2.441 | 0.557 | -4.425 | -1.235 |
| OTU20075 | -2.629 | 0.333 | -4.313 | -1.928 |
| OTU22342 | -2.536 | 0.595 | -4.590 | -1.135 |
| OTU21636 | -2.700 | 0.324 | -4.258 | -1.962 |
| OTU20097 | -2.760 | 0.273 | -4.323 | -2.173 |
| OTU17491 | -2.705 | 0.475 | -4.368 | -1.615 |
| OTU18932 | -2.751 | 0.328 | -4.070 | -1.931 |
| OTU24696 | -2.760 | 0.429 | -4.240 | -1.639 |
| OTU17740 | -2.639 | 0.666 | -4.434 | -1.228 |
| OTU26865 | -2.805 | 0.325 | -4.289 | -2.115 |
| OTU20442 | -2.823 | 0.409 | -4.226 | -1.518 |
| OTU22207 | -2.921 | 0.350 | -4.261 | -2.000 |
| OTU23028 | -2.769 | 0.464 | -4.400 | -1.615 |
| OTU14860 | -2.963 | 0.368 | -4.288 | -2.218 |
| OTU16767 | -3.035 | 0.356 | -4.260 | -2.298 |
| OTU28397 | -2.973 | 0.376 | -4.220 | -2.001 |
| OTU15300 | -2.863 | 0.543 | -4.437 | -1.558 |
| OTU18390 | -3.063 | 0.410 | -4.403 | -2.171 |
| OTU19694 | -2.995 | 0.395 | -4.388 | -2.202 |
| OTU25269 | -2.762 | 0.677 | -4.456 | -1.383 |
| OTU26116 | -2.829 | 0.630 | -4.476 | -1.237 |
| OTU28603 | -3.051 | 0.376 | -4.199 | -2.170 |
| OTU28842 | -2.990 | 0.440 | -4.502 | -1.999 |
| OTU33579 | -3.076 | 0.379 | -4.349 | -2.342 |
| OTU16090 | -2.817 | 0.646 | -4.562 | -1.379 |
| OTU15957 | -3.067 | 0.535 | -4.535 | -1.864 |
| OTU17350 | -3.062 | 0.415 | -4.336 | -2.131 |
| OTU24985 | -3.172 | 0.346 | -4.327 | -2.271 |
| OTU25379 | -3.076 | 0.480 | -4.528 | -1.656 |

| | | | | |
|---|---|---|---|---|
| OTU27979 | -3.212 | 0.353 | -4.371 | -2.413 |
| OTU30658 | -2.938 | 0.601 | -4.419 | -1.648 |
| OTU33243 | -3.188 | 0.306 | -4.310 | -2.552 |
| OTU35558 | -3.092 | 0.354 | -4.227 | -2.206 |
| OTU14099 | -2.971 | 0.617 | -4.769 | -1.588 |
| OTU5148 | -3.370 | 0.327 | -4.337 | -2.442 |
| OTU9731 | -3.335 | 0.412 | -4.471 | -2.322 |
| OTU11339 | -3.015 | 0.651 | -4.498 | -1.473 |
| OTU12689 | -3.347 | 0.381 | -4.427 | -2.406 |
| OTU14730 | -3.095 | 0.622 | -4.514 | -1.710 |
| OTU15709 | -3.286 | 0.368 | -4.328 | -2.503 |
| OTU17032 | -3.305 | 0.409 | -4.383 | -2.333 |
| OTU18150 | -3.309 | 0.342 | -4.348 | -2.562 |
| OTU19325 | -3.062 | 0.604 | -4.582 | -1.415 |
| OTU19623 | -3.368 | 0.402 | -4.358 | -2.460 |
| OTU20333 | -3.038 | 0.693 | -4.681 | -1.403 |
| OTU20576 | -3.432 | 0.321 | -4.408 | -2.803 |
| OTU22045 | -3.004 | 0.627 | -4.456 | -1.619 |
| OTU22363 | -3.147 | 0.568 | -4.578 | -1.711 |
| OTU23005 | -3.011 | 0.627 | -4.699 | -1.663 |
| OTU23606 | -3.204 | 0.471 | -4.419 | -1.934 |
| OTU23975 | -3.237 | 0.432 | -4.558 | -2.221 |
| OTU24715 | -3.188 | 0.484 | -4.472 | -1.954 |
| OTU27116 | -3.203 | 0.428 | -4.510 | -2.240 |
| OTU28205 | -3.262 | 0.447 | -4.320 | -2.075 |
| OTU28969 | -3.253 | 0.478 | -4.484 | -2.160 |
| OTU32093 | -3.333 | 0.390 | -4.353 | -2.412 |
| OTU32230 | -3.219 | 0.448 | -4.560 | -2.148 |
| OTU33421 | -3.327 | 0.365 | -4.375 | -2.418 |
| OTU33984 | -3.276 | 0.424 | -4.439 | -2.343 |
| OTU35368 | -3.393 | 0.388 | -4.297 | -2.526 |
| OTU35998 | -3.266 | 0.424 | -4.361 | -2.272 |
| OTU36484 | -3.148 | 0.532 | -4.471 | -1.886 |
| OTU36691 | -3.187 | 0.390 | -4.275 | -2.273 |
| OTU40042 | -3.246 | 0.420 | -4.593 | -2.328 |
| OTU42388 | -3.287 | 0.465 | -4.447 | -2.122 |
| OTU3615 | -2.999 | 0.815 | -4.980 | -0.820 |
| OTU10057 | -3.299 | 0.513 | -4.599 | -1.938 |
| OTU13989 | -3.540 | 0.377 | -4.514 | -2.672 |
| OTU14011 | -3.538 | 0.343 | -4.411 | -2.760 |
| OTU14750 | -3.478 | 0.453 | -4.514 | -2.351 |
| OTU15028 | -3.479 | 0.530 | -4.539 | -2.006 |
| OTU15766 | -3.391 | 0.444 | -4.469 | -2.337 |
| OTU16297 | -3.065 | 0.798 | -4.627 | -1.169 |
| OTU17060 | -3.553 | 0.384 | -4.422 | -2.436 |

| | | | | |
|---|---|---|---|---|
| OTU17889 | -3.469 | 0.396 | -4.573 | -2.401 |
| OTU17986 | -3.596 | 0.373 | -4.446 | -2.687 |
| OTU19048 | -3.496 | 0.378 | -4.509 | -2.503 |
| OTU19337 | -3.457 | 0.362 | -4.520 | -2.621 |
| OTU20456 | -3.307 | 0.632 | -4.587 | -1.526 |
| OTU20718 | -3.563 | 0.423 | -4.538 | -2.536 |
| OTU21089 | -3.358 | 0.581 | -4.705 | -2.014 |
| OTU21103 | -3.379 | 0.499 | -4.730 | -1.975 |
| OTU21572 | -3.554 | 0.438 | -4.432 | -2.454 |
| OTU21685 | -3.331 | 0.488 | -4.638 | -1.826 |
| OTU21738 | -3.556 | 0.406 | -4.578 | -2.603 |
| OTU22931 | -3.444 | 0.403 | -4.501 | -2.499 |
| OTU23082 | -3.410 | 0.442 | -4.466 | -2.240 |
| OTU23089 | -2.989 | 0.734 | -5.150 | -0.967 |
| OTU23189 | -3.425 | 0.528 | -4.511 | -1.962 |
| OTU23411 | -3.281 | 0.639 | -4.606 | -1.665 |
| OTU23692 | -3.290 | 0.593 | -4.592 | -1.914 |
| OTU24471 | -3.417 | 0.505 | -4.491 | -2.214 |
| OTU24562 | -3.182 | 0.696 | -4.849 | -1.387 |
| OTU24722 | -3.628 | 0.368 | -4.444 | -2.739 |
| OTU24936 | -3.236 | 0.658 | -4.646 | -1.697 |
| OTU25483 | -3.383 | 0.542 | -4.410 | -2.172 |
| OTU25795 | -3.349 | 0.565 | -4.585 | -2.151 |
| OTU26092 | -3.190 | 0.675 | -5.179 | -1.360 |
| OTU26118 | -3.331 | 0.616 | -4.903 | -1.609 |
| OTU26847 | -3.323 | 0.505 | -4.577 | -1.825 |
| OTU27145 | -3.534 | 0.393 | -4.420 | -2.612 |
| OTU27257 | -3.185 | 0.599 | -4.627 | -1.828 |
| OTU28290 | -3.359 | 0.553 | -4.635 | -1.982 |
| OTU28553 | -3.599 | 0.360 | -4.659 | -2.687 |
| OTU28557 | -3.597 | 0.368 | -4.470 | -2.726 |
| OTU28882 | -3.481 | 0.373 | -4.386 | -2.606 |
| OTU28957 | -3.267 | 0.563 | -4.735 | -1.579 |
| OTU28965 | -3.171 | 0.546 | -4.730 | -1.544 |
| OTU29084 | -3.476 | 0.403 | -4.474 | -2.488 |
| OTU29342 | -3.489 | 0.454 | -4.504 | -2.422 |
| OTU29519 | -3.447 | 0.390 | -4.318 | -2.530 |
| OTU29609 | -3.584 | 0.397 | -4.502 | -2.447 |
| OTU29627 | -3.421 | 0.509 | -4.424 | -2.236 |
| OTU30089 | -3.472 | 0.455 | -4.459 | -2.400 |
| OTU30111 | -3.595 | 0.356 | -4.508 | -2.421 |
| OTU30174 | -3.214 | 0.525 | -4.511 | -1.923 |
| OTU30840 | -3.338 | 0.456 | -4.496 | -2.111 |
| OTU31095 | -3.407 | 0.389 | -4.580 | -2.470 |
| OTU32740 | -3.508 | 0.428 | -4.533 | -2.512 |

| | | | | |
|---|---|---|---|---|
| OTU33382 | -3.619 | 0.377 | -4.467 | -2.660 |
| OTU33451 | -3.572 | 0.404 | -4.423 | -2.467 |
| OTU33466 | -3.298 | 0.515 | -4.592 | -1.900 |
| OTU33934 | -3.489 | 0.456 | -4.469 | -2.339 |
| OTU34075 | -3.493 | 0.479 | -4.753 | -2.026 |
| OTU34721 | -3.599 | 0.379 | -4.535 | -2.582 |
| OTU34852 | -3.395 | 0.404 | -4.464 | -2.376 |
| OTU35400 | -3.424 | 0.357 | -4.445 | -2.606 |
| OTU35548 | -3.474 | 0.393 | -4.478 | -2.521 |
| OTU35875 | -3.486 | 0.390 | -4.544 | -2.453 |
| OTU35889 | -3.274 | 0.546 | -4.519 | -2.029 |
| OTU35979 | -3.540 | 0.430 | -4.465 | -2.312 |
| OTU36501 | -3.467 | 0.446 | -4.610 | -2.399 |
| OTU36628 | -3.354 | 0.471 | -4.424 | -2.218 |
| OTU36970 | -3.394 | 0.433 | -4.344 | -2.282 |
| OTU37029 | -3.611 | 0.361 | -4.476 | -2.696 |
| OTU37543 | -3.548 | 0.362 | -4.383 | -2.643 |
| OTU39601 | -3.469 | 0.456 | -4.639 | -2.236 |
| OTU40229 | -3.584 | 0.340 | -4.366 | -2.804 |
| OTU40602 | -3.658 | 0.333 | -4.513 | -2.696 |
| OTU40690 | -3.596 | 0.405 | -4.581 | -2.532 |
| OTU40724 | -3.558 | 0.439 | -4.606 | -2.451 |
| OTU41353 | -3.447 | 0.404 | -4.458 | -2.402 |
| OTU41913 | -3.409 | 0.369 | -4.455 | -2.569 |
| OTU43368 | -3.622 | 0.379 | -4.495 | -2.431 |
| OTU46742 | -3.648 | 0.366 | -4.425 | -2.660 |
| OTU47648 | -3.531 | 0.442 | -4.379 | -2.214 |
| OTU76611 | -3.567 | 0.433 | -4.532 | -2.522 |
