# Supplementary material for: Host genetics and diet, but not immunoglobulin A expression, converge to shape compositional features of the gut microbiome in an advanced intercross population of mice
Source: Genome Biol. 2014 Dec 17;15(12):552. doi: 10.1186/s13059-014-0552-6 (PMC4290092; doi:10.1186/s13059-014-0552-6)
Supplement: Additional file 2: — Table showing the contributions of four variance components to the total variation in the abundances of the 203 microbiota taxa. [file 13059_2014_552_MOESM2_ESM.pdf]

Additional file 2. Estimates of four random components (cohort, family, litter, and residual) and their proportion of the total variation, for each of the 203 microbiota taxa.

	Variance Components				% of Total Variation			
Taxon	Cohort	Family	Litter	Residual	Cohort	Family	Parity	Residual
Alistipes	0.0003	0.0000	0.0008	0.0247	1.30	0.00	3.24	95.46
Bacteroides	0.0112	0.0013	0.0073	0.0578	14.46	1.63	9.46	74.45
Parabacteroides	0.0082	0.0069	0.0021	0.0659	9.89	8.32	2.56	79.23
Helicobacter	0.0082	0.0072	0.0075	0.1219	5.68	4.97	5.16	84.19
Oscillibacter	0.0008	0.0000	0.0044	0.0492	1.40	0.00	8.04	90.56
Odoribacter	0.0161	0.0019	0.0062	0.0475	22.51	2.60	8.60	66.29
Dorea	0.0059	0.0041	0.0054	0.0452	9.76	6.80	8.95	74.48
Lactococcus	0.0386	0.0029	0.0010	0.1200	23.77	1.76	0.60	73.86
Lactobacillus	0.0538	0.0034	0.0000	0.4223	11.21	0.72	0.00	88.07
Mucispirillum	0.0134	0.0186	0.0217	0.3226	3.57	4.93	5.77	85.73
TM7_genera_incertae_sedis	0.0650	0.0000	0.0924	0.3004	14.20	0.00	20.18	65.62
Ureaplasma	0.1073	0.0544	0.0402	0.3002	21.37	10.83	8.00	59.80
Roseburia	0.1059	0.0022	0.0000	0.2018	34.16	0.71	0.00	65.13
Butyricicoccus	0.0460	0.0082	0.0451	0.2239	14.23	2.52	13.95	69.29
Turicibacter	0.0653	0.0347	0.0000	0.6429	8.78	4.68	0.00	86.54
Rikenella	0.0657	0.0412	0.0226	0.4245	11.86	7.44	4.08	76.62
Alistipes_OTU15	0.0003	0.0000	0.0039	0.0294	0.81	0.00	11.62	87.57
Alistipes_OTU13	0.0006	0.0001	0.0000	0.0297	1.82	0.27	0.00	97.91
Bacteroides_OTU3	0.0122	0.0000	0.0112	0.1099	9.13	0.00	8.40	82.47
HelicobacterganmaniT	0.0059	0.0084	0.0150	0.1540	3.22	4.61	8.18	83.99
Oscillibacter_OTU3	0.0018	0.0004	0.0050	0.0517	3.07	0.67	8.45	87.81
Parabacteroides_OTU3	0.0114	0.0173	0.0276	0.1563	5.36	8.13	12.98	73.53
Parabacteroides_OTU6	0.0018	0.0000	0.0892	0.3831	0.38	0.00	18.81	80.81
Odoribacter_OTU6	0.0147	0.0029	0.0039	0.0490	20.85	4.10	5.53	69.51
Bacteroides_OTU12	0.0455	0.0723	0.0000	0.2970	10.97	17.44	0.00	71.59
Parabacteroides_OTU2	0.0299	0.0139	0.0003	0.1653	14.28	6.62	0.15	78.95
Dorea_OTU7	0.0068	0.0000	0.0071	0.0537	10.10	0.00	10.52	79.38
LactMG1363	0.0399	0.0010	0.0032	0.1312	22.74	0.59	1.82	74.85
Bacteroides_OTU13	0.0211	0.0079	0.0248	0.1572	10.02	3.76	11.74	74.48
Lactobacillusapodemit	0.0683	0.0000	0.0000	0.4052	14.43	0.00	0.00	85.57
Odoribacter_OTU1	0.0076	0.0000	0.0102	0.0684	8.81	0.00	11.79	79.40
Mucispirillumschaedlerit	0.0134	0.0186	0.0217	0.3226	3.57	4.93	5.77	85.73
Dorea_OTU12	0.0368	0.0352	0.0166	0.1653	14.47	13.88	6.55	65.10
Bacteroides_OTU0	0.0158	0.0000	0.0007	0.1872	7.75	0.00	0.36	91.89
Oscillibacter_OTU14	0.0000	0.0060	0.0125	0.1504	0.00	3.55	7.41	89.04
UreaplasmaurealyticumT	0.1198	0.0362	0.0512	0.2839	24.39	7.37	10.43	57.81
LactococcusSK11	0.0491	0.0095	0.0000	0.1610	22.36	4.34	0.00	73.30
Alistipes_OTU2	0.0199	0.0056	0.0063	0.1425	11.41	3.22	3.62	81.76
Helicobacter51449	0.0125	0.0482	0.0000	0.2770	3.69	14.28	0.00	82.02

Oscillibacter_OTU6	0.0361	0.0346	0.0142	0.2297	11.47	10.99	4.52	73.03
Butyricicoccus_OTU7	0.0485	0.0069	0.0484	0.2298	14.55	2.08	14.51	68.86
Roseburia_OTU7	0.1799	0.0087	0.0000	0.2276	43.22	2.09	0.00	54.69
Lactobacillus33200	0.0630	0.0541	0.0405	0.5581	8.80	7.56	5.65	77.98
TM7_genera_incertae_sedis_OTU143	0.0436	0.0140	0.0543	0.2717	11.37	3.65	14.15	70.83
Parabacteroides_OTU8	0.0152	0.0013	0.0186	0.1338	8.99	0.78	11.04	79.20
Parabacteroides_OTU9	0.0182	0.0000	0.0078	0.1509	10.28	0.00	4.40	85.31
Odoribacter_OTU2	0.1468	0.0242	0.0638	0.1831	35.13	5.80	15.26	43.82
Bacteroides_OTU7	0.0456	0.0108	0.0111	0.3360	11.30	2.67	2.76	83.27
OTU4825	0.0053	0.0000	0.0034	0.1054	4.66	0.00	2.96	92.38
OTU12405	0.0159	0.0072	0.0000	0.0606	19.03	8.64	0.00	72.33
OTU13263	0.0082	0.0000	0.0055	0.1001	7.18	0.00	4.84	87.98
OTU10148	0.0024	0.0445	0.0171	0.1412	1.15	21.68	8.34	68.83
OTU20373	0.0020	0.0000	0.0054	0.0824	2.21	0.00	6.00	91.79
OTU26285	0.0346	0.0048	0.0041	0.1477	18.07	2.53	2.16	77.24
OTU20360	0.0327	0.0081	0.0334	0.1428	15.07	3.74	15.37	65.82
OTU27073	0.0924	0.0120	0.0000	0.2033	30.01	3.91	0.00	66.08
OTU21224	0.0141	0.0000	0.0342	0.2953	4.11	0.00	9.94	85.94
OTU20075	0.0168	0.0000	0.0056	0.1101	12.65	0.00	4.22	83.13
OTU22342	0.0786	0.0000	0.0809	0.3008	17.07	0.00	17.59	65.35
OTU21636	0.0031	0.0090	0.0003	0.1032	2.66	7.80	0.22	89.32
OTU20097	0.0014	0.0023	0.0021	0.0741	1.79	2.92	2.69	92.61
OTU17491	0.0206	0.0455	0.0290	0.1739	7.65	16.92	10.78	64.65
OTU18932	0.0100	0.0000	0.0123	0.1048	7.89	0.00	9.67	82.44
OTU24696	0.0025	0.0398	0.0148	0.1499	1.23	19.22	7.16	72.39
OTU17740	0.0319	0.0590	0.0182	0.3957	6.33	11.68	3.60	78.39
OTU26865	0.0235	0.0000	0.0062	0.1044	17.51	0.00	4.65	77.84
OTU20442	0.0389	0.0202	0.0218	0.1420	17.46	9.05	9.78	63.71
OTU22207	0.0297	0.0154	0.0034	0.1123	18.49	9.55	2.11	69.85
OTU23028	0.0199	0.0440	0.0182	0.1721	7.83	17.30	7.17	67.70
OTU14860	0.0192	0.0064	0.0009	0.1345	11.94	3.97	0.56	83.54
OTU16767	0.0032	0.0123	0.0074	0.1145	2.34	8.93	5.37	83.36
OTU28397	0.0058	0.0016	0.0041	0.1412	3.82	1.02	2.71	92.45
OTU15300	0.0131	0.0000	0.0316	0.2815	4.02	0.00	9.68	86.30
OTU18390	0.0080	0.0042	0.0179	0.1561	4.30	2.27	9.61	83.82
OTU19694	0.0096	0.0090	0.0016	0.1529	5.53	5.20	0.90	88.38
OTU25269	0.0193	0.0684	0.0171	0.4041	3.79	13.44	3.36	79.41
OTU26116	0.0288	0.0406	0.0057	0.3748	6.41	9.03	1.27	83.29
OTU28603	0.0141	0.0069	0.0017	0.1400	8.65	4.26	1.04	86.04
OTU28842	0.0635	0.0000	0.0306	0.1753	23.55	0.00	11.37	65.08
OTU33579	0.0085	0.0039	0.0022	0.1440	5.34	2.47	1.36	90.83
OTU16090	0.0697	0.0703	0.0322	0.3522	13.30	13.40	6.14	67.17
OTU15957	0.0133	0.0000	0.0357	0.2688	4.18	0.00	11.25	84.58
OTU17350	0.0095	0.0127	0.0183	0.1548	4.84	6.49	9.38	79.29

OTU24985	0.0022	0.0000	0.0125	0.1153	1.69	0.00	9.60	88.72
OTU25379	0.0346	0.0158	0.0218	0.2086	12.31	5.62	7.75	74.31
OTU27979	0.0163	0.0000	0.0000	0.1286	11.24	0.00	0.00	88.76
OTU30658	0.0338	0.0601	0.0337	0.3011	7.87	14.03	7.87	70.24
OTU33243	0.0010	0.0000	0.0094	0.0899	0.97	0.00	9.41	89.61
OTU35558	0.0111	0.0000	0.0040	0.1271	7.82	0.00	2.78	89.40
OTU14099	0.0698	0.0572	0.0351	0.3236	14.38	11.77	7.22	66.62
OTU5148	0.0293	0.0000	0.0061	0.1059	20.73	0.00	4.32	74.96
OTU9731	0.0176	0.0000	0.0286	0.1539	8.79	0.00	14.30	76.91
OTU11339	0.0495	0.0363	0.0000	0.4110	9.96	7.31	0.00	82.74
OTU12689	0.0149	0.0025	0.0142	0.1372	8.83	1.48	8.41	81.29
OTU14730	0.0738	0.0473	0.0337	0.3385	14.97	9.59	6.83	68.61
OTU15709	0.0028	0.0074	0.0115	0.1257	1.89	5.00	7.78	85.33
OTU17032	0.0051	0.0482	0.0000	0.1366	2.70	25.38	0.00	71.92
OTU18150	0.0029	0.0006	0.0095	0.1132	2.26	0.49	7.54	89.71
OTU19325	0.0405	0.0285	0.0127	0.3450	9.50	6.67	2.99	80.84
OTU19623	0.0040	0.0099	0.0054	0.1547	2.28	5.67	3.08	88.97
OTU20333	0.0057	0.0000	0.0963	0.4182	1.10	0.00	18.51	80.39
OTU20576	0.0149	0.0046	0.0000	0.1024	12.22	3.76	0.00	84.01
OTU22045	0.0688	0.0550	0.0596	0.3192	13.69	10.95	11.85	63.50
OTU22363	0.0321	0.0093	0.0302	0.3032	8.57	2.48	8.05	80.89
OTU23005	0.0904	0.0512	0.0263	0.3459	17.59	9.96	5.12	67.33
OTU23606	0.0207	0.0425	0.0307	0.1708	7.83	16.06	11.61	64.50
OTU23975	0.0235	0.0000	0.0090	0.1860	10.77	0.00	4.11	85.12
OTU24715	0.0102	0.0000	0.0222	0.2250	3.95	0.00	8.64	87.41
OTU27116	0.0355	0.0499	0.0048	0.1493	14.81	20.84	2.00	62.35
OTU28205	0.0000	0.0068	0.0366	0.1693	0.00	3.22	17.22	79.56
OTU28969	0.0063	0.0112	0.0219	0.2099	2.51	4.49	8.80	84.20
OTU32093	0.0118	0.0000	0.0192	0.1427	6.78	0.00	11.07	82.15
OTU32230	0.0157	0.0123	0.0047	0.1949	6.90	5.40	2.05	85.65
OTU33421	0.0000	0.0030	0.0056	0.1299	0.00	2.17	4.06	93.77
OTU33984	0.0057	0.0012	0.0147	0.1731	2.91	0.64	7.57	88.89
OTU35368	0.0000	0.0000	0.0107	0.1454	0.00	0.00	6.85	93.15
OTU35998	0.0150	0.0000	0.0130	0.1754	7.37	0.00	6.41	86.22
OTU36484	0.0566	0.0097	0.0298	0.2634	15.75	2.71	8.28	73.26
OTU36691	0.0391	0.0092	0.0000	0.1511	19.62	4.63	0.00	75.75
OTU40042	0.0225	0.0173	0.0150	0.1560	10.67	8.19	7.11	74.03
OTU42388	0.0096	0.0051	0.0000	0.2199	4.07	2.15	0.00	93.77
OTU3615	0.0700	0.1454	0.0756	0.5360	8.47	17.58	9.14	64.82
OTU10057	0.0418	0.0332	0.0091	0.2404	12.89	10.23	2.79	74.09
OTU13989	0.0001	0.0060	0.0000	0.1416	0.06	4.05	0.00	95.89
OTU14011	0.0148	0.0000	0.0012	0.1203	10.85	0.00	0.91	88.24
OTU14750	0.0045	0.0037	0.0120	0.1999	2.05	1.70	5.44	90.81
OTU15028	0.0140	0.0313	0.0000	0.2679	4.47	10.01	0.00	85.52
OTU15766	0.0165	0.0227	0.0067	0.1845	7.18	9.85	2.90	80.08

OTU16297	0.0784	0.0478	0.1566	0.5048	9.96	6.07	19.88	64.09
OTU17060	0.0069	0.0000	0.0085	0.1453	4.27	0.00	5.30	90.43
OTU17889	0.0304	0.0197	0.0000	0.1476	15.37	9.97	0.00	74.66
OTU17986	0.0064	0.0000	0.0137	0.1322	4.19	0.00	9.02	86.79
OTU19048	0.0046	0.0156	0.0070	0.1311	2.88	9.84	4.43	82.85
OTU19337	0.0093	0.0014	0.0025	0.1326	6.38	0.94	1.71	90.97
OTU20456	0.0000	0.0026	0.0976	0.3308	0.00	0.61	22.65	76.74
OTU20718	0.0048	0.0266	0.0302	0.1378	2.42	13.32	15.17	69.09
OTU21089	0.0479	0.0449	0.0307	0.2885	11.63	10.90	7.45	70.02
OTU21103	0.0301	0.0025	0.0251	0.2351	10.29	0.86	8.57	80.29
OTU21572	0.0146	0.0069	0.0179	0.1777	6.73	3.19	8.24	81.83
OTU21685	0.0203	0.0106	0.0000	0.2368	7.57	3.95	0.00	88.47
OTU21738	0.0241	0.0124	0.0087	0.1536	12.10	6.26	4.39	77.24
OTU22931	0.0174	0.0125	0.0042	0.1544	9.25	6.64	2.22	81.89
OTU23082	0.0093	0.0055	0.0165	0.1855	4.31	2.52	7.61	85.56
OTU23089	0.0893	0.0283	0.0634	0.4806	13.50	4.28	9.59	72.64
OTU23189	0.0085	0.0374	0.0000	0.2627	2.76	12.12	0.00	85.12
OTU23411	0.0081	0.0000	0.0697	0.3657	1.82	0.00	15.71	82.47
OTU23692	0.0324	0.0491	0.0320	0.3008	7.82	11.85	7.72	72.61
OTU24471	0.0042	0.0143	0.0345	0.2272	1.49	5.09	12.31	81.11
OTU24562	0.1247	0.0370	0.0221	0.4530	19.59	5.80	3.47	71.14
OTU24722	0.0018	0.0060	0.0020	0.1336	1.26	4.19	1.41	93.14
OTU24936	0.0100	0.0000	0.0738	0.3879	2.11	0.00	15.64	82.25
OTU25483	0.0061	0.0450	0.0000	0.2683	1.91	14.09	0.00	84.00
OTU25795	0.0167	0.0448	0.0298	0.2734	4.58	12.29	8.17	74.97
OTU26092	0.1147	0.0071	0.0794	0.4003	19.07	1.19	13.19	66.55
OTU26118	0.0373	0.0189	0.0401	0.3433	8.49	4.31	9.12	78.08
OTU26847	0.0625	0.0135	0.0124	0.2435	18.83	4.08	3.75	73.34
OTU27145	0.0092	0.0024	0.0137	0.1464	5.36	1.43	7.96	85.25
OTU27257	0.0426	0.0538	0.0332	0.3055	9.79	12.36	7.62	70.22
OTU28290	0.0210	0.0181	0.0181	0.2910	6.04	5.20	5.19	83.57
OTU28553	0.0000	0.0052	0.0024	0.1279	0.00	3.82	1.81	94.37
OTU28557	0.0040	0.0052	0.0169	0.1218	2.71	3.49	11.42	82.38
OTU28882	0.0084	0.0000	0.0041	0.1414	5.47	0.00	2.67	91.86
OTU28957	0.1092	0.0000	0.0242	0.3082	24.74	0.00	5.48	69.79
OTU28965	0.1398	0.0607	0.0201	0.2421	30.22	13.12	4.35	52.32
OTU29084	0.0218	0.0094	0.0000	0.1612	11.35	4.89	0.00	83.76
OTU29342	0.0022	0.0277	0.0359	0.1649	0.95	12.00	15.54	71.50
OTU29519	0.0061	0.0130	0.0000	0.1482	3.67	7.79	0.00	88.55
OTU29609	0.0342	0.0000	0.0172	0.1488	17.08	0.00	8.57	74.34
OTU29627	0.0000	0.0000	0.0075	0.2607	0.00	0.00	2.81	97.19
OTU30089	0.0007	0.0233	0.0390	0.1668	0.30	10.16	16.96	72.58
OTU30111	0.0422	0.0033	0.0055	0.1250	23.97	1.85	3.12	71.06
OTU30174	0.0887	0.0112	0.0190	0.2607	23.36	2.95	5.01	68.67
OTU30840	0.0370	0.0000	0.0249	0.1946	14.44	0.00	9.69	75.87

OTU31095	0.1059	0.0008	0.0180	0.1419	39.72	0.29	6.75	53.23
OTU32740	0.0293	0.0000	0.0127	0.1794	13.23	0.00	5.73	81.04
OTU33382	0.0127	0.0000	0.0000	0.1473	7.96	0.00	0.00	92.04
OTU33451	0.0124	0.0093	0.0146	0.1502	6.65	4.97	7.81	80.57
OTU33466	0.0154	0.0069	0.0089	0.2626	5.23	2.34	3.02	89.41
OTU33934	0.0250	0.0036	0.0235	0.1933	10.18	1.45	9.56	78.81
OTU34075	0.0124	0.0075	0.0099	0.2233	4.90	2.98	3.90	88.22
OTU34721	0.0184	0.0078	0.0025	0.1402	10.92	4.61	1.48	83.00
OTU34852	0.0557	0.0010	0.0079	0.1615	24.64	0.45	3.49	71.43
OTU35400	0.0419	0.0015	0.0000	0.1313	23.99	0.85	0.02	75.13
OTU35548	0.0092	0.0022	0.0088	0.1509	5.35	1.30	5.16	88.19
OTU35875	0.0130	0.0024	0.0010	0.1556	7.57	1.41	0.59	90.43
OTU35889	0.0162	0.0212	0.0056	0.2890	4.87	6.40	1.67	87.06
OTU35979	0.0050	0.0110	0.0242	0.1626	2.47	5.41	11.95	80.16
OTU36501	0.0323	0.0092	0.0099	0.1912	13.33	3.78	4.08	78.81
OTU36628	0.0206	0.0131	0.0217	0.2027	7.98	5.06	8.39	78.56
OTU36970	0.0109	0.0217	0.0158	0.1648	5.09	10.19	7.41	77.31
OTU37029	0.0129	0.0068	0.0078	0.1231	8.58	4.53	5.19	81.70
OTU37543	0.0062	0.0094	0.0000	0.1282	4.31	6.55	0.00	89.14
OTU39601	0.0370	0.0063	0.0056	0.2065	14.50	2.47	2.20	80.83
OTU40229	0.0052	0.0000	0.0041	0.1159	4.16	0.00	3.29	92.55
OTU40602	0.0216	0.0010	0.0049	0.1100	15.74	0.70	3.57	80.00
OTU40690	0.0334	0.0320	0.0000	0.1426	16.06	15.40	0.00	68.54
OTU40724	0.0228	0.0000	0.0135	0.1889	10.11	0.00	6.00	83.90
OTU41353	0.0214	0.0146	0.0000	0.1572	11.08	7.54	0.00	81.38
OTU41913	0.0366	0.0000	0.0036	0.1376	20.59	0.00	2.00	77.41
OTU43368	0.0181	0.0045	0.0029	0.1427	10.74	2.68	1.74	84.84
OTU46742	0.0107	0.0095	0.0004	0.1320	7.00	6.22	0.26	86.52
OTU47648	0.0072	0.0048	0.0064	0.1937	3.39	2.26	3.03	91.32
OTU76611	0.0126	0.0059	0.0227	0.1717	5.92	2.79	10.65	80.64
