# Supplementary figures and images for: Host genetics and diet, but not immunoglobulin A expression, converge to shape compositional features of the gut microbiome in an advanced intercross population of mice
Source: Genome Biol. 2014 Dec 17;15(12):552. doi: 10.1186/s13059-014-0552-6 (PMC4290092; doi:10.1186/s13059-014-0552-6)

# G4

Bacteroidetes  
Proteobacteria  
Actinobacteria  
Firmicutes

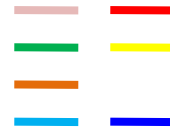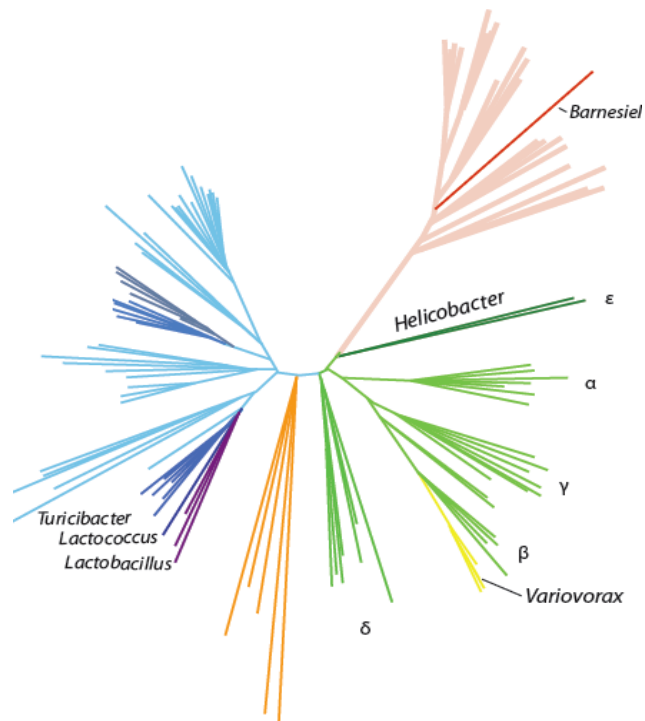

# G10

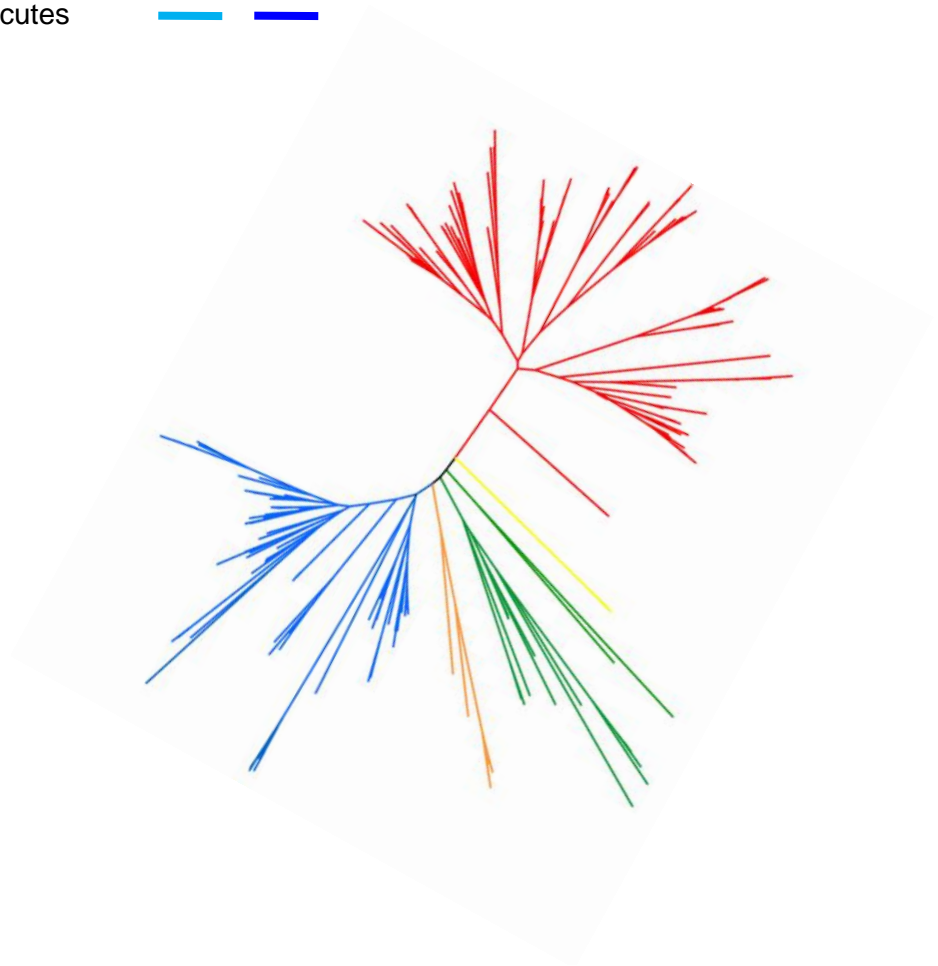

Supplement: Additional file 3: — Figure illustrating a phylogenetic analysis of the microbiota taxa abundances in the G 4 and G 10 mouse intercross generations. [file 13059_2014_552_MOESM3_ESM.pdf]
