# Supplementary material for: Host genetics and diet, but not immunoglobulin A expression, converge to shape compositional features of the gut microbiome in an advanced intercross population of mice
Source: Genome Biol. 2014 Dec 17;15(12):552. doi: 10.1186/s13059-014-0552-6 (PMC4290092; doi:10.1186/s13059-014-0552-6)
Supplement: Additional file 4: — Table showing the basic statistics for the G4 taxa processed through the same OTU pipeline as the G10 taxa. [file 13059_2014_552_MOESM4_ESM.pdf]

| Rank | Taxon | Mean | Median | Standard D | Minimum | Maximum |
|---|---|---|---|---|---|---|
| phylum | *Actinobacteria* | -3.19057 | -3.18984 | 0.614641 | -4.64687 | -0.46494 |
| phylum | *Bacteroidetes* | -1.20611 | -1.1447 | 0.411206 | -2.83603 | -0.36688 |
| phylum | *Cyanobacteria* | -3.09911 | -3.20276 | 0.826736 | -4.4516 | -0.46906 |
| phylum | *Firmicutes* | -0.83769 | -0.86166 | 0.298357 | -1.7675 | -0.10447 |
| phylum | *Proteobacteria* | -1.6144 | -1.55853 | 0.46635 | -4.054 | -0.22595 |
| phylum | *TM7* | -2.40866 | -2.29094 | 0.59878 | -4.27678 | -1.18612 |
| class | *Actinobacteria* | -3.19057 | -3.18984 | 0.614641 | -4.64687 | -0.46494 |
| class | *Alphaproteobacteria* | -3.42676 | -3.52627 | 0.618384 | -4.64687 | -1.64374 |
| class | *Bacilli* | -1.23417 | -1.23771 | 0.520657 | -2.48579 | -0.10626 |
| class | *Bacteroidia* | -1.21015 | -1.14994 | 0.41212 | -2.87079 | -0.3676 |
| class | *Betaproteobacteria* | -2.40094 | -2.38145 | 0.500164 | -4.054 | -0.54811 |
| class | *Clostridia* | -1.30633 | -1.28025 | 0.213934 | -2.49186 | -0.70922 |
| class | *Cyanobacteria* | -3.09911 | -3.20276 | 0.826736 | -4.4516 | -0.46906 |
| class | *Epsilonproteobacteria* | -2.20364 | -1.88716 | 0.956221 | -4.64687 | -0.51214 |
| class | *Erysipelotrichi* | -2.49994 | -2.54562 | 0.877167 | -4.55945 | -0.42552 |
| class | *Gammaproteobacteria* | -2.63435 | -2.58818 | 0.653046 | -4.26221 | -0.24354 |
| order | *Bacillales* | -3.08396 | -3.11361 | 0.633737 | -4.36256 | -0.69915 |
| order | *Bacteroidales* | -1.21015 | -1.14994 | 0.41212 | -2.87079 | -0.3676 |
| order | *Burkholderiales* | -2.42552 | -2.40386 | 0.509457 | -4.054 | -0.56194 |
| order | *Campylobacterales* | -2.20364 | -1.88716 | 0.956221 | -4.64687 | -0.51214 |
| order | *Clostridiales* | -1.30634 | -1.28025 | 0.213936 | -2.49186 | -0.70922 |
| order | *Enterobacteriales* | -3.36754 | -3.35358 | 0.547795 | -4.64687 | -1.5487 |
| order | *Erysipelotrichales* | -2.49994 | -2.54562 | 0.877167 | -4.55945 | -0.42552 |
| order | *Lactobacillales* | -1.25191 | -1.2597 | 0.522845 | -2.51879 | -0.10626 |
| order | *Pseudomonadales* | -2.97615 | -2.95049 | 0.607164 | -4.4516 | -0.24354 |
| family | *Bacteroidaceae* | -1.84075 | -1.78637 | 0.539524 | -4.25101 | -0.56328 |
| family | *Chloroplast* | -3.0997 | -3.20276 | 0.827561 | -4.60249 | -0.46906 |
| family | *Comamonadaceae* | -2.57645 | -2.56674 | 0.517346 | -4.054 | -0.57141 |
| family | *Enterobacteriaceae* | -3.36754 | -3.35358 | 0.547795 | -4.64687 | -1.5487 |
| family | *Erysipelotrichaceae* | -2.49994 | -2.54562 | 0.877167 | -4.55945 | -0.42552 |
| family | *Helicobacteraceae* | -2.2174 | -1.88767 | 0.973223 | -4.64687 | -0.51214 |
| family | *Incertae Sedis XIII* | -3.44172 | -3.39076 | 0.421002 | -4.54888 | -2.39368 |
| family | *Lachnospiraceae* | -1.75276 | -1.73965 | 0.261692 | -3.03593 | -0.97874 |
| family | *Lactobacillaceae* | -1.74941 | -1.78549 | 0.710664 | -4.08408 | -0.10741 |
| family | *Leuconostocaceae* | -2.69641 | -2.60737 | 0.570624 | -4.4567 | -1.14245 |
| family | *Moraxellaceae* | -3.27338 | -3.23729 | 0.556482 | -4.4567 | -1.80805 |
| family | *Porphyromonadaceae* | -1.65123 | -1.55873 | 0.461944 | -4.25101 | -0.68982 |
| family | *Pseudomonadaceae* | -3.33827 | -3.33036 | 0.653186 | -4.60249 | -0.24379 |
| family | *Rikenellaceae* | -1.74743 | -1.69201 | 0.405664 | -3.13704 | -0.9474 |
| family | *Ruminococcaceae* | -1.58681 | -1.54628 | 0.273405 | -3.04013 | -0.71772 |
| family | *Staphylococcaceae* | -3.28371 | -3.35558 | 0.697969 | -4.57636 | -0.69915 |
| family | *Streptococcaceae* | -1.7532 | -1.84831 | 0.586326 | -3.2864 | -0.27968 |
| genus | *Acidovorax* | -3.46804 | -3.49721 | 0.568918 | -4.48199 | -1.44669 |
| genus | *Acinetobacter* | -3.2981 | -3.26345 | 0.557951 | -4.4567 | -1.81114 |
| genus | *Alistipes* | -1.80916 | -1.76913 | 0.418428 | -3.15619 | -0.96023 |
| genus | *Anaerovorax* | -3.4424 | -3.39076 | 0.421483 | -4.54888 | -2.39368 |

| | | | | | | |
|---|---|---|---|---|---|---|
| genus | *Bacteroides* | -1.84075 | -1.78637 | 0.539524 | -4.25101 | -0.56328 |
| genus | *Butyricicoccus* | -3.12847 | -3.08934 | 0.547655 | -4.38396 | -1.89672 |
| genus | *Dorea* | -1.87215 | -1.84829 | 0.290798 | -3.03593 | -1.00098 |
| genus | *Helicobacter* | -2.21755 | -1.88767 | 0.973256 | -4.64687 | -0.51214 |
| genus | *Lactobacillus* | -1.74941 | -1.78549 | 0.710664 | -4.08408 | -0.10741 |
| genus | *Lactococcus* | -1.76753 | -1.85944 | 0.593581 | -3.2864 | -0.28022 |
| genus | *Leuconostoc* | -3.37483 | -3.33594 | 0.491983 | -4.6313 | -1.97467 |
| genus | *Odoribacter* | -2.25905 | -2.20892 | 0.472891 | -4.25101 | -1.31754 |
| genus | *Oscillibacter* | -1.63201 | -1.58462 | 0.288863 | -3.52827 | -1.07909 |
| genus | *Parabacteroides* | -1.81992 | -1.74717 | 0.500228 | -4.25101 | -0.6969 |
| genus | *Pseudomonas* | -3.33867 | -3.33036 | 0.653305 | -4.60249 | -0.24379 |
| genus | *Rikenella* | -3.05039 | -2.83105 | 0.783282 | -4.55945 | -1.60787 |
| genus | *Roseburia* | -2.64471 | -2.59433 | 0.434967 | -4.25667 | -1.45208 |
| genus | *Streptococcus* | -3.54715 | -3.55121 | 0.524559 | -4.64687 | -1.39531 |
| genus | *Streptophyta* | -3.09971 | -3.20276 | 0.827555 | -4.60249 | -0.46906 |
| genus | *TM7_genera_incertae_sedis* | -2.40866 | -2.29094 | 0.59878 | -4.27678 | -1.18612 |
| genus | *Turicibacter* | -2.71959 | -2.78561 | 1.030603 | -4.55945 | -0.4258 |
| genus | *Variovorax* | -2.82857 | -2.84029 | 0.579754 | -4.25101 | -0.64626 |
| genus | *Weissella* | -2.83751 | -2.70329 | 0.639479 | -4.64687 | -1.20845 |
| species | *Acinetobacter junii SH205* | -3.47148 | -3.50978 | 0.567486 | -4.6313 | -1.83342 |
| species | *Alistipes_OTU11* | -2.11161 | -2.05525 | 0.427259 | -3.85516 | -1.26842 |
| species | *Alistipes_OTU9* | -2.14951 | -2.13978 | 0.46028 | -4.23533 | -1.21399 |
| species | *Anaerovorax odorimutans (T)* | -3.44869 | -3.40295 | 0.421989 | -4.54888 | -2.39368 |
| species | *Bacteroides_OTU1* | -2.78441 | -2.67251 | 0.961569 | -4.64687 | -0.76623 |
| species | *Bacteroides_OTU17* | -2.49121 | -2.46785 | 0.691637 | -4.30612 | -0.77056 |
| species | *Bacteroides_OTU21* | -2.71022 | -2.67348 | 0.61911 | -4.50226 | -0.68252 |
| species | *Butyricicoccus_OTU6* | -3.15399 | -3.1154 | 0.564175 | -4.51092 | -1.89672 |
| species | *Dorea longicatena DSM 13814* | -2.36784 | -2.35411 | 0.311116 | -3.83658 | -1.46842 |
| species | *Helicobacter ganmani (T)* | -2.23827 | -1.90919 | 0.972735 | -4.64687 | -0.51426 |
| species | *Lactobacillus apodemi (T)* | -2.00552 | -1.99539 | 0.749607 | -4.21548 | -0.2863 |
| species | *Lactobacillus johnsonii ATCC 3[illegible]* | -2.80012 | -2.90586 | 0.953665 | -4.54888 | -0.10779 |
| species | *Lactococcus lactis subsp. crem[illegible]* | -2.07914 | -2.12129 | 0.672315 | -3.90466 | -0.50728 |
| species | *Lactococcus lactis subsp. crem[illegible]* | -2.18527 | -2.24201 | 0.599832 | -4.03214 | -0.53762 |
| species | *Leuconostoc citreum KM20* | -3.37557 | -3.33594 | 0.49168 | -4.6313 | -1.97467 |
| species | *Odoribacter_OTU3* | -2.85187 | -2.76297 | 0.598281 | -4.37723 | -1.64085 |
| species | *Odoribacter_OTU6* | -2.45372 | -2.39076 | 0.453557 | -4.25101 | -1.62771 |
| species | *Oscillibacter_OTU18* | -2.84293 | -2.78661 | 0.445496 | -4.41929 | -1.71246 |
| species | *Oscillibacter_OTU5* | -1.70635 | -1.66359 | 0.296221 | -3.52827 | -1.0981 |
| species | *Oscillibacter_OTU8* | -3.22396 | -3.13194 | 0.64276 | -4.4567 | -1.71814 |
| species | *OTU10059* | -3.61574 | -3.58482 | 0.717842 | -4.78302 | -2.04879 |
| species | *OTU100635* | -4.07641 | -4.40085 | 0.644617 | -4.9479 | -1.994 |
| species | *OTU10180* | -3.80517 | -3.83658 | 0.648205 | -4.81195 | -2.25018 |
| species | *OTU10250* | -3.87864 | -4.37519 | 0.871435 | -4.85926 | -1.27116 |
| species | *OTU10810* | -4.04452 | -4.40688 | 0.739492 | -4.93233 | -1.27015 |
| species | *OTU10815* | -3.47717 | -3.40083 | 0.742625 | -4.84001 | -2.04756 |
| species | *OTU109685* | -4.07846 | -4.40078 | 0.636706 | -5.02378 | -1.51063 |
| species | *OTU111345* | -3.61488 | -3.53129 | 0.653347 | -4.90352 | -1.78221 |

| | | | | | | |
|---|---|---|---|---|---|---|
| species | *OTU11309* | -3.36035 | -3.2837 | 0.757491 | -4.87739 | -1.40144 |
| species | *OTU11573* | -3.10033 | -2.94052 | 0.74066 | -4.93233 | -1.71972 |
| species | *OTU118435* | -3.7291 | -3.7759 | 0.681505 | -4.9479 | -1.83989 |
| species | *OTU119723* | -3.89061 | -3.99463 | 0.635751 | -4.9479 | -2.20326 |
| species | *OTU12637* | -4.12298 | -4.39263 | 0.605222 | -5.02378 | -1.9065 |
| species | *OTU12884* | -4.01975 | -4.35141 | 0.651102 | -5.02378 | -1.99758 |
| species | *OTU12948* | -3.62006 | -3.52264 | 0.488057 | -4.7489 | -2.53283 |
| species | *OTU1311* | -3.34027 | -3.25237 | 0.828055 | -4.75773 | -1.36371 |
| species | *OTU1370* | -3.74091 | -3.74241 | 0.604181 | -5.02378 | -2.27746 |
| species | *OTU14515* | -2.88147 | -2.48592 | 1.214826 | -4.93233 | -0.99988 |
| species | *OTU14778* | -2.3405 | -2.2071 | 0.770515 | -4.87739 | -0.81285 |
| species | *OTU15542* | -3.52757 | -3.43008 | 0.590226 | -4.6973 | -1.81561 |
| species | *OTU15599* | -3.98079 | -4.35395 | 0.6772 | -4.9479 | -2.34901 |
| species | *OTU15621* | -2.72934 | -2.63188 | 0.759325 | -4.72032 | -0.82946 |
| species | *OTU15852* | -2.9158 | -2.77163 | 0.754775 | -4.9479 | -1.53102 |
| species | *OTU16644* | -3.57069 | -3.50379 | 0.631004 | -4.77273 | -2.0539 |
| species | *OTU16771* | -3.77041 | -3.78704 | 0.627962 | -4.90352 | -2.34286 |
| species | *OTU16907* | -3.34571 | -3.25565 | 0.76087 | -4.93233 | -1.98368 |
| species | *OTU17214* | -3.64056 | -3.59244 | 0.705112 | -4.9479 | -1.88235 |
| species | *OTU17277* | -4.12783 | -4.41704 | 0.705146 | -5.02378 | -1.42246 |
| species | *OTU17532* | -3.23373 | -3.1594 | 0.772274 | -4.9479 | -1.57487 |
| species | *OTU1754* | -3.65547 | -3.6185 | 0.620907 | -4.84001 | -2.07064 |
| species | *OTU17916* | -3.59306 | -3.55121 | 0.635098 | -4.7489 | -2.03914 |
| species | *OTU18194* | -3.93396 | -4.04135 | 0.601667 | -4.78302 | -2.05809 |
| species | *OTU18550* | -2.8939 | -2.69556 | 0.720274 | -4.7489 | -1.51658 |
| species | *OTU18933* | -3.50712 | -3.41577 | 0.6534 | -5.02378 | -2.00116 |
| species | *OTU19064* | -3.60713 | -3.64157 | 0.814385 | -4.86048 | -1.54217 |
| species | *OTU19089* | -3.85027 | -3.999 | 0.69254 | -4.9479 | -1.79934 |
| species | *OTU19169* | -3.51471 | -3.56324 | 0.839679 | -4.86048 | -1.43294 |
| species | *OTU19497* | -3.73166 | -3.77934 | 0.656358 | -4.9479 | -1.61958 |
| species | *OTU20339* | -2.3626 | -2.26317 | 0.737521 | -4.66626 | -0.98974 |
| species | *OTU20360* | -2.80858 | -2.59988 | 0.746414 | -4.93233 | -1.5156 |
| species | *OTU20550* | -3.84391 | -3.88247 | 0.648093 | -4.85926 | -2.34425 |
| species | *OTU20553* | -2.90934 | -2.67025 | 0.910885 | -4.87739 | -1.28408 |
| species | *OTU20580* | -3.42148 | -3.3934 | 0.86792 | -4.86048 | -1.33443 |
| species | *OTU20628* | -3.99287 | -4.2999 | 0.623813 | -4.9479 | -2.29122 |
| species | *OTU21345* | -2.86668 | -2.75051 | 0.720151 | -4.9479 | -1.28267 |
| species | *OTU21486* | -3.33413 | -3.15874 | 0.696693 | -4.9479 | -2.08784 |
| species | *OTU21516* | -4.1548 | -4.42015 | 0.653733 | -4.93233 | -1.55403 |
| species | *OTU21517* | -3.93222 | -4.03222 | 0.604007 | -4.9479 | -1.94417 |
| species | *OTU21756* | -3.65664 | -3.56225 | 0.589912 | -4.86048 | -2.37057 |
| species | *OTU22095* | -3.72566 | -3.73388 | 0.646545 | -4.9479 | -2.14825 |
| species | *OTU22302* | -3.79149 | -4.32976 | 0.94491 | -4.93233 | -1.07396 |
| species | *OTU22570* | -3.11864 | -2.94349 | 0.655318 | -4.78302 | -1.72283 |
| species | *OTU22574* | -3.40792 | -3.28001 | 0.590209 | -4.81195 | -2.2072 |
| species | *OTU22618* | -3.43432 | -3.31709 | 0.72649 | -4.93233 | -1.33829 |
| species | *OTU22876* | -3.35517 | -3.25334 | 0.753412 | -4.9479 | -1.43361 |

| | | | | | | |
|---|---|---|---|---|---|---|
| species | *OTU22892* | -3.59662 | -3.62675 | 0.768 | -4.9479 | -1.72987 |
| species | *OTU23162* | -3.8867 | -3.92547 | 0.610727 | -4.87739 | -2.24698 |
| species | *OTU23399* | -3.01818 | -3.02001 | 0.95746 | -5.02378 | -1.36247 |
| species | *OTU23401* | -3.26045 | -2.96209 | 0.944632 | -4.9479 | -1.52655 |
| species | *OTU23520* | -3.4137 | -3.27852 | 0.647696 | -4.81195 | -1.46343 |
| species | *OTU23521* | -3.14677 | -3.07151 | 0.51531 | -4.76337 | -2.03668 |
| species | *OTU23591* | -4.35042 | -4.44765 | 0.46547 | -5.02378 | -1.21209 |
| species | *OTU23632* | -4.05169 | -4.34084 | 0.589542 | -4.9479 | -2.18221 |
| species | *OTU23893* | -3.24796 | -3.10358 | 0.665633 | -4.9479 | -1.90283 |
| species | *OTU23901* | -3.6434 | -3.76065 | 0.753222 | -4.86048 | -1.89245 |
| species | *OTU24957* | -3.16799 | -3.08975 | 0.607516 | -4.78302 | -1.82906 |
| species | *OTU25398* | -3.51036 | -3.40572 | 0.70716 | -4.9479 | -1.98126 |
| species | *OTU25443* | -3.94077 | -4.27903 | 0.656551 | -4.93233 | -2.18915 |
| species | *OTU255658* | -4.05042 | -4.35472 | 0.618173 | -5.02378 | -1.48086 |
| species | *OTU25567* | -3.57916 | -3.49087 | 0.602017 | -4.9479 | -2.16777 |
| species | *OTU25580* | -2.79023 | -2.68931 | 0.622751 | -4.66359 | -1.49569 |
| species | *OTU26078* | -3.53094 | -3.51182 | 0.793481 | -4.87739 | -1.51721 |
| species | *OTU26150* | -3.84625 | -3.85205 | 0.593466 | -4.80419 | -2.27425 |
| species | *OTU26207* | -4.00827 | -4.29164 | 0.608823 | -4.86048 | -2.09954 |
| species | *OTU26335* | -3.96149 | -4.19667 | 0.615594 | -4.9479 | -1.95059 |
| species | *OTU26562* | -2.75857 | -2.64591 | 0.56831 | -4.70253 | -1.49097 |
| species | *OTU26691* | -2.72722 | -2.55042 | 0.852519 | -4.72032 | -0.95849 |
| species | *OTU2681* | -3.65527 | -3.65475 | 0.702151 | -4.93233 | -1.97387 |
| species | *OTU26919* | -3.76705 | -3.75686 | 0.563221 | -4.76337 | -2.3891 |
| species | *OTU26927* | -3.11909 | -3.20119 | 1.092727 | -4.9479 | -0.41051 |
| species | *OTU26940* | -3.15281 | -2.99191 | 0.646928 | -4.85926 | -2.01859 |
| species | *OTU2714* | -4.12539 | -4.3927 | 0.603126 | -5.02378 | -1.69682 |
| species | *OTU27559* | -2.53373 | -2.44503 | 0.488059 | -4.45649 | -1.62167 |
| species | *OTU27668* | -3.37379 | -3.20875 | 0.672856 | -4.7489 | -1.98997 |
| species | *OTU27929* | -3.95829 | -4.31998 | 0.703523 | -4.93233 | -1.83902 |
| species | *OTU2796* | -3.60507 | -3.54452 | 0.684644 | -4.80329 | -1.75514 |
| species | *OTU27993* | -4.14132 | -4.41491 | 0.601863 | -4.93233 | -1.9853 |
| species | *OTU28031* | -3.70418 | -3.76492 | 0.692849 | -4.81195 | -1.85716 |
| species | *OTU28127* | -4.29004 | -4.4456 | 0.545393 | -4.9479 | -1.26747 |
| species | *OTU28357* | -3.24393 | -3.10243 | 0.770966 | -4.9479 | -1.68626 |
| species | *OTU28363* | -3.85874 | -3.87823 | 0.593836 | -4.93233 | -2.35147 |
| species | *OTU28378* | -3.75889 | -3.7813 | 0.568076 | -4.87739 | -2.3553 |
| species | *OTU28641* | -4.12113 | -4.40078 | 0.618598 | -5.02378 | -1.84753 |
| species | *OTU28763* | -3.4433 | -3.40346 | 0.790509 | -4.9479 | -1.37227 |
| species | *OTU28844* | -3.58532 | -3.48151 | 0.609641 | -4.85926 | -2.37692 |
| species | *OTU28881* | -3.2315 | -3.13814 | 0.544248 | -4.74457 | -2.04546 |
| species | *OTU29136* | -3.50764 | -3.42234 | 0.638403 | -4.90352 | -2.18115 |
| species | *OTU29205* | -3.45279 | -3.3534 | 0.649163 | -4.87739 | -2.15138 |
| species | *OTU2939* | -3.63674 | -3.56423 | 0.813222 | -5.02378 | -1.89534 |
| species | *OTU29411* | -4.01363 | -4.3204 | 0.63103 | -5.02378 | -2.21073 |
| species | *OTU29481* | -3.35032 | -3.10659 | 0.823864 | -4.80329 | -1.76289 |
| species | *OTU29497* | -3.87503 | -3.8512 | 0.567573 | -4.9479 | -2.43026 |

| | | | | | | |
|---|---|---|---|---|---|---|
| species | *OTU29676* | -3.74965 | -3.6936 | 0.534053 | -4.84001 | -2.57394 |
| species | *OTU29690* | -3.54901 | -3.45962 | 0.586855 | -4.9479 | -2.05237 |
| species | *OTU29993* | -3.73957 | -3.67495 | 0.510351 | -4.80329 | -2.33186 |
| species | *OTU30239* | -3.42912 | -3.26521 | 0.612642 | -4.7489 | -2.1839 |
| species | *OTU30406* | -3.0865 | -2.98265 | 0.616838 | -4.84991 | -1.75124 |
| species | *OTU30440* | -3.86468 | -3.91698 | 0.646418 | -4.84001 | -1.78511 |
| species | *OTU30466* | -3.23159 | -3.17493 | 0.658556 | -4.7489 | -1.33681 |
| species | *OTU30575* | -3.80856 | -3.80168 | 0.583403 | -5.02378 | -2.3544 |
| species | *OTU30906* | -2.98125 | -2.8544 | 0.796941 | -4.78302 | -1.32542 |
| species | *OTU30974* | -3.68024 | -3.74593 | 0.754539 | -4.78302 | -2.0299 |
| species | *OTU31161* | -3.64097 | -3.64128 | 0.763066 | -4.90352 | -1.71433 |
| species | *OTU31225* | -3.47844 | -3.47849 | 0.929298 | -5.02378 | -1.44989 |
| species | *OTU31270* | -3.0741 | -2.90719 | 0.776111 | -4.87739 | -1.5814 |
| species | *OTU31437* | -3.73291 | -3.74547 | 0.60667 | -4.9479 | -2.3149 |
| species | *OTU31812* | -4.13251 | -4.38511 | 0.584323 | -4.9479 | -1.72024 |
| species | *OTU31854* | -3.57316 | -3.53832 | 0.72164 | -4.90352 | -2.04173 |
| species | *OTU31861* | -3.73152 | -3.81211 | 0.732957 | -4.85926 | -2.18349 |
| species | *OTU31900* | -3.00706 | -2.91966 | 0.874355 | -4.78302 | -1.27646 |
| species | *OTU32146* | -3.038 | -2.8273 | 0.852627 | -4.75773 | -1.53971 |
| species | *OTU32159* | -3.91542 | -4.04036 | 0.629538 | -4.86048 | -2.19348 |
| species | *OTU32201* | -3.77034 | -3.87691 | 0.71626 | -4.9479 | -1.56969 |
| species | *OTU32261* | -3.61593 | -3.50037 | 0.557959 | -4.81195 | -2.34311 |
| species | *OTU32556* | -4.08321 | -4.3898 | 0.670964 | -5.02378 | -1.49192 |
| species | *OTU32850* | -3.66376 | -3.57692 | 0.569555 | -4.84001 | -2.28661 |
| species | *OTU32953* | -3.37257 | -3.24487 | 0.680441 | -4.87739 | -1.88289 |
| species | *OTU33094* | -3.74891 | -3.7402 | 0.646007 | -4.85926 | -2.29057 |
| species | *OTU33106* | -4.22365 | -4.43258 | 0.542139 | -5.02378 | -2.07748 |
| species | *OTU33162* | -3.20696 | -3.08114 | 0.724066 | -4.9479 | -1.55419 |
| species | *OTU3343* | -3.73043 | -4.13033 | 0.922542 | -4.9479 | -1.41134 |
| species | *OTU33766* | -3.91775 | -4.05805 | 0.615579 | -5.02378 | -1.95685 |
| species | *OTU33959* | -3.89364 | -3.92932 | 0.560359 | -4.9479 | -2.2363 |
| species | *OTU33981* | -3.29276 | -3.18427 | 0.596602 | -4.7489 | -2.01747 |
| species | *OTU33995* | -3.92046 | -4.19667 | 0.6733 | -4.87739 | -2.01986 |
| species | *OTU34250* | -3.18362 | -3.08938 | 0.608237 | -4.7489 | -1.59082 |
| species | *OTU34564* | -3.24586 | -3.13155 | 0.747958 | -4.7489 | -1.56467 |
| species | *OTU34602* | -3.24108 | -3.1567 | 0.613171 | -4.72032 | -1.73563 |
| species | *OTU34676* | -3.89853 | -4.13033 | 0.655265 | -4.9479 | -2.36433 |
| species | *OTU34910* | -3.68677 | -3.61023 | 0.477362 | -4.7489 | -2.54375 |
| species | *OTU34980* | -3.25223 | -3.13672 | 0.58693 | -4.90352 | -2.05999 |
| species | *OTU35123* | -3.60426 | -3.49471 | 0.560047 | -4.85926 | -2.49066 |
| species | *OTU35132* | -3.31682 | -3.18549 | 0.600323 | -4.75773 | -2.12905 |
| species | *OTU35474* | -3.95614 | -4.24797 | 0.625175 | -4.93233 | -2.35683 |
| species | *OTU35522* | -3.53283 | -3.43867 | 0.638097 | -4.93233 | -2.24805 |
| species | *OTU35653* | -3.64353 | -3.7608 | 0.825302 | -5.02378 | -1.98934 |
| species | *OTU35851* | -3.79157 | -3.89087 | 0.731348 | -4.9479 | -2.19667 |
| species | *OTU36357* | -3.77066 | -3.67495 | 0.58071 | -4.93233 | -2.50292 |
| species | *OTU3637* | -4.01307 | -4.40688 | 0.767714 | -4.93233 | -1.40201 |

| | | | | | | |
|---|---|---|---|---|---|---|
| species | *OTU36453* | -4.01822 | -4.37011 | 0.6995 | -5.02378 | -1.66937 |
| species | *OTU36913* | -3.18825 | -3.12937 | 1.003814 | -4.93233 | -1.38186 |
| species | *OTU36973* | -4.22648 | -4.43014 | 0.580442 | -5.02378 | -1.50455 |
| species | *OTU37155* | -3.44059 | -3.36091 | 0.565092 | -4.93233 | -1.93846 |
| species | *OTU37416* | -3.90053 | -3.92381 | 0.62554 | -4.9479 | -0.73644 |
| species | *OTU37511* | -3.64833 | -3.83487 | 0.898265 | -5.02378 | -1.72442 |
| species | *OTU37550* | -4.20592 | -4.43527 | 0.648013 | -5.02378 | -1.37345 |
| species | *OTU37576* | -3.31094 | -3.21212 | 0.873071 | -4.87739 | -1.49066 |
| species | *OTU37609* | -3.86534 | -3.95775 | 0.666795 | -5.02378 | -2.46379 |
| species | *OTU37848* | -3.9184 | -4.23573 | 0.680585 | -4.90352 | -2.04456 |
| species | *OTU38053* | -3.39192 | -3.27076 | 0.596936 | -4.80419 | -2.17082 |
| species | *OTU38425* | -4.02817 | -4.35311 | 0.636688 | -5.02378 | -2.34439 |
| species | *OTU38701* | -3.70682 | -3.63251 | 0.578008 | -4.85926 | -2.37139 |
| species | *OTU38736* | -3.88688 | -4.27035 | 0.713231 | -4.9479 | -2.02835 |
| species | *OTU390* | -3.64143 | -3.8014 | 0.837914 | -4.9479 | -1.76297 |
| species | *OTU39205* | -2.92025 | -2.81258 | 0.547235 | -4.67162 | -1.68994 |
| species | *OTU39325* | -3.12088 | -3.00828 | 0.604102 | -4.70449 | -0.70851 |
| species | *OTU39477* | -3.77117 | -3.79064 | 0.659269 | -4.85926 | -2.25219 |
| species | *OTU39918* | -3.94471 | -4.15537 | 0.609506 | -4.93233 | -2.10206 |
| species | *OTU40040* | -3.50302 | -3.41902 | 0.613551 | -4.80419 | -2.0829 |
| species | *OTU40052* | -3.67417 | -3.73143 | 0.729797 | -4.87739 | -1.89998 |
| species | *OTU40057* | -3.81531 | -3.94171 | 0.689111 | -4.84991 | -2.02474 |
| species | *OTU40238* | -3.29668 | -3.22515 | 0.7378 | -4.76337 | -1.82328 |
| species | *OTU40448* | -3.10196 | -2.94789 | 0.616058 | -4.84991 | -1.77589 |
| species | *OTU4046* | -3.71931 | -3.71834 | 0.677688 | -4.81195 | -2.26293 |
| species | *OTU40540* | -2.69623 | -2.47337 | 0.905389 | -4.70862 | -0.99188 |
| species | *OTU40685* | -3.38004 | -3.33696 | 0.823593 | -4.9479 | -1.55725 |
| species | *OTU40859* | -3.6243 | -3.62944 | 0.715622 | -4.85926 | -1.92627 |
| species | *OTU40878* | -3.7845 | -3.94998 | 0.759877 | -4.93233 | -1.85676 |
| species | *OTU40993* | -2.8506 | -2.46419 | 0.968362 | -4.75773 | -1.46839 |
| species | *OTU41413* | -3.79492 | -3.78774 | 0.53448 | -4.93233 | -1.92942 |
| species | *OTU41518* | -3.19363 | -3.0475 | 0.685503 | -4.9479 | -1.8352 |
| species | *OTU41577* | -3.79665 | -3.765 | 0.553101 | -4.9479 | -2.66564 |
| species | *OTU4201* | -3.45517 | -3.77952 | 1.0806 | -5.02378 | -0.8168 |
| species | *OTU42081* | -3.85709 | -3.98159 | 0.669124 | -4.9479 | -2.2796 |
| species | *OTU43081* | -3.76787 | -3.88502 | 0.751234 | -5.02378 | -1.97187 |
| species | *OTU43451* | -3.84267 | -3.89697 | 0.608082 | -4.93233 | -2.4725 |
| species | *OTU43477* | -3.96388 | -4.29164 | 0.651112 | -4.9479 | -2.21465 |
| species | *OTU43491* | -3.48437 | -3.38855 | 0.571829 | -4.80419 | -2.06127 |
| species | *OTU43960* | -3.94839 | -4.34994 | 0.747613 | -5.02378 | -1.77452 |
| species | *OTU44212* | -3.05412 | -2.95122 | 0.607526 | -4.81195 | -1.96136 |
| species | *OTU44783* | -3.68508 | -3.63448 | 0.593634 | -4.81195 | -1.93064 |
| species | *OTU44879* | -3.51178 | -3.39432 | 0.549508 | -4.74457 | -2.51818 |
| species | *OTU44911* | -3.65449 | -3.63367 | 0.684188 | -4.85926 | -1.84608 |
| species | *OTU45365* | -3.83212 | -3.86213 | 0.646681 | -4.87739 | -1.8133 |
| species | *OTU45531* | -3.81868 | -4.38187 | 0.92046 | -5.02378 | -1.31226 |
| species | *OTU46032* | -3.86375 | -3.86106 | 0.550942 | -4.87739 | -2.11492 |

| | | | | | | |
|---|---|---|---|---|---|---|
| species | *OTU4608* | -2.61585 | -2.47581 | 0.58345 | -4.72032 | -1.45611 |
| species | *OTU46167* | -3.52575 | -3.42754 | 0.591575 | -4.84001 | -2.24652 |
| species | *OTU4622* | -2.47759 | -2.30151 | 0.888438 | -4.84991 | -1.05422 |
| species | *OTU46295* | -3.73663 | -3.80325 | 0.654183 | -4.84991 | -1.94495 |
| species | *OTU47045* | -3.82296 | -3.79463 | 0.543541 | -4.85926 | -2.44742 |
| species | *OTU47283* | -3.85166 | -3.93817 | 0.654563 | -4.85926 | -2.44299 |
| species | *OTU4729* | -3.9816 | -4.33349 | 0.707726 | -5.02378 | -1.56301 |
| species | *OTU47332* | -3.84041 | -3.8739 | 0.629218 | -5.02378 | -2.20687 |
| species | *OTU47335* | -3.52882 | -3.4553 | 0.530817 | -4.84001 | -2.27078 |
| species | *OTU47724* | -3.60262 | -3.53218 | 0.711178 | -4.9479 | -1.87198 |
| species | *OTU47830* | -2.94411 | -2.78524 | 0.685899 | -4.72032 | -1.62878 |
| species | *OTU47858* | -3.71849 | -3.73878 | 0.661954 | -4.84991 | -1.83285 |
| species | *OTU47964* | -4.14405 | -4.40511 | 0.604741 | -5.02378 | -1.78696 |
| species | *OTU48141* | -3.978 | -4.28421 | 0.619076 | -5.02378 | -2.11481 |
| species | *OTU48279* | -3.46551 | -3.38044 | 0.594879 | -4.81195 | -1.88642 |
| species | *OTU48293* | -3.91691 | -4.02111 | 0.6282 | -4.86048 | -1.97947 |
| species | *OTU48302* | -3.7995 | -3.90249 | 0.715377 | -4.81195 | -1.65595 |
| species | *OTU48397* | -4.08056 | -4.38187 | 0.601058 | -5.02378 | -1.73083 |
| species | *OTU48521* | -3.21676 | -3.13071 | 0.683828 | -4.7489 | -1.7911 |
| species | *OTU48892* | -4.24127 | -4.4187 | 0.474913 | -4.9479 | -1.72209 |
| species | *OTU49049* | -3.6728 | -3.60686 | 0.616179 | -4.81195 | -2.26831 |
| species | *OTU49635* | -3.89556 | -4.01708 | 0.630044 | -4.87739 | -2.24797 |
| species | *OTU50422* | -3.46763 | -3.3894 | 0.806388 | -5.02378 | -1.53662 |
| species | *OTU50512* | -3.89834 | -3.95564 | 0.605167 | -4.85926 | -2.02538 |
| species | *OTU50609* | -3.6449 | -3.59813 | 0.710401 | -4.75773 | -1.66815 |
| species | *OTU50790* | -3.42756 | -3.22819 | 0.782239 | -5.02378 | -1.95984 |
| species | *OTU51156* | -3.65241 | -3.65475 | 0.688257 | -4.9479 | -1.77815 |
| species | *OTU51177* | -3.78468 | -3.74032 | 0.567668 | -4.9479 | -2.43726 |
| species | *OTU51188* | -3.97972 | -4.23573 | 0.601488 | -4.93233 | -2.3934 |
| species | *OTU51268* | -3.21541 | -3.12024 | 0.58978 | -4.72032 | -1.90756 |
| species | *OTU51413* | -3.80468 | -4.18096 | 0.793088 | -4.85926 | -1.56846 |
| species | *OTU51595* | -3.51988 | -3.46923 | 0.726169 | -4.80419 | -1.96709 |
| species | *OTU51780* | -3.73252 | -3.71834 | 0.576674 | -4.7489 | -2.28105 |
| species | *OTU52100* | -3.77739 | -4.24797 | 0.862564 | -4.90352 | -1.62182 |
| species | *OTU52368* | -3.60365 | -3.537 | 0.709856 | -4.9479 | -2.18568 |
| species | *OTU52370* | -3.48029 | -3.45182 | 0.500951 | -4.87739 | -2.16118 |
| species | *OTU5244* | -3.79193 | -3.83321 | 0.647931 | -4.9479 | -2.14768 |
| species | *OTU52658* | -3.76459 | -3.97183 | 0.813113 | -4.9479 | -1.09956 |
| species | *OTU52864* | -3.5465 | -3.46845 | 0.599881 | -4.75924 | -2.11047 |
| species | *OTU53206* | -3.7561 | -3.68538 | 0.515651 | -4.85926 | -2.64054 |
| species | *OTU53228* | -3.59263 | -3.48567 | 0.576189 | -4.85926 | -2.44167 |
| species | *OTU53441* | -3.43098 | -3.32341 | 0.7733 | -5.02378 | -1.73293 |
| species | *OTU53615* | -3.84518 | -3.85908 | 0.623009 | -4.87739 | -2.37149 |
| species | *OTU53737* | -4.08508 | -4.39347 | 0.661046 | -5.02378 | -1.87913 |
| species | *OTU54291* | -3.63853 | -3.59021 | 0.679109 | -4.7489 | -1.86363 |
| species | *OTU54456* | -3.86127 | -3.88213 | 0.638364 | -5.02378 | -2.24538 |
| species | *OTU55494* | -3.93366 | -4.09258 | 0.589764 | -4.81195 | -2.31226 |

| | | | | | | |
|---|---|---|---|---|---|---|
| species | *OTU55752* | -3.70299 | -3.6249 | 0.50715 | -4.75773 | -2.53111 |
| species | *OTU55854* | -3.72472 | -3.62997 | 0.574821 | -4.90352 | -2.54449 |
| species | *OTU55866* | -3.99966 | -4.31998 | 0.640823 | -5.02378 | -1.89202 |
| species | *OTU56222* | -3.75756 | -4.14854 | 0.864235 | -4.90352 | -1.58095 |
| species | *OTU5650* | -3.87106 | -3.92018 | 0.65405 | -4.9479 | -1.89101 |
| species | *OTU56636* | -3.92057 | -3.93485 | 0.5911 | -5.02378 | -1.87252 |
| species | *OTU5675* | -3.73636 | -3.73227 | 0.593979 | -4.85926 | -2.40632 |
| species | *OTU57343* | -4.07361 | -4.3757 | 0.654209 | -5.02378 | -1.60107 |
| species | *OTU5750* | -2.71951 | -2.60894 | 0.609623 | -4.54664 | -1.49624 |
| species | *OTU57537* | -3.84248 | -3.83052 | 0.567734 | -4.93233 | -2.06487 |
| species | *OTU5790* | -4.08314 | -4.39396 | 0.666728 | -5.02378 | -1.65761 |
| species | *OTU57920* | -3.89121 | -3.92865 | 0.603534 | -5.02378 | -2.18973 |
| species | *OTU58156* | -3.72255 | -3.64591 | 0.512914 | -4.75924 | -2.6268 |
| species | *OTU58813* | -3.46127 | -3.39433 | 0.933313 | -5.02378 | -1.8313 |
| species | *OTU58877* | -3.66801 | -3.54648 | 0.539731 | -4.78302 | -2.38392 |
| species | *OTU6000* | -3.77794 | -3.78704 | 0.615655 | -4.9479 | -2.40149 |
| species | *OTU60504* | -3.87908 | -4.10243 | 0.690201 | -4.9479 | -1.90535 |
| species | *OTU6068* | -4.07046 | -4.38108 | 0.650533 | -4.9479 | -1.79934 |
| species | *OTU62137* | -3.7648 | -4.05477 | 0.810655 | -5.02378 | -1.45354 |
| species | *OTU62773* | -3.81988 | -3.78774 | 0.552473 | -4.9479 | -2.49771 |
| species | *OTU6284* | -3.48253 | -3.62762 | 0.973652 | -5.02378 | -0.61434 |
| species | *OTU6324* | -4.34604 | -4.44267 | 0.37454 | -5.02378 | -1.93008 |
| species | *OTU6326* | -3.93773 | -4.20672 | 0.662662 | -4.93233 | -2.202 |
| species | *OTU63993* | -4.32668 | -4.44932 | 0.502719 | -4.9479 | -1.58552 |
| species | *OTU64213* | -3.74468 | -3.75686 | 0.634961 | -4.9479 | -1.75996 |
| species | *OTU64589* | -4.40202 | -4.45326 | 0.309712 | -5.02378 | -1.83018 |
| species | *OTU65403* | -4.01618 | -4.35079 | 0.647713 | -5.02378 | -2.27394 |
| species | *OTU65505* | -4.41942 | -4.46359 | 0.375163 | -5.02378 | -1.41195 |
| species | *OTU66878* | -3.92443 | -4.01774 | 0.600743 | -4.90352 | -2.25856 |
| species | *OTU67167* | -4.2077 | -4.43476 | 0.620536 | -5.02378 | -1.75072 |
| species | *OTU6836* | -3.86914 | -4.10992 | 0.680885 | -4.9479 | -2.14752 |
| species | *OTU68552* | -3.91766 | -4.27533 | 0.676627 | -4.84001 | -2.05529 |
| species | *OTU68927* | -3.95034 | -4.31437 | 0.681689 | -5.02378 | -1.97061 |
| species | *OTU699* | -2.7175 | -2.57095 | 0.857761 | -4.9479 | -1.1064 |
| species | *OTU7027* | -2.91933 | -2.84029 | 0.518671 | -4.7489 | -1.93077 |
| species | *OTU70446* | -4.1151 | -4.41737 | 0.658577 | -5.02378 | -1.62378 |
| species | *OTU70876* | -3.8707 | -4.10721 | 0.708993 | -4.9479 | -1.82137 |
| species | *OTU72339* | -4.11082 | -4.40085 | 0.596713 | -5.02378 | -2.1489 |
| species | *OTU72899* | -3.72933 | -3.81258 | 0.70777 | -5.02378 | -2.03794 |
| species | *OTU7358* | -3.83094 | -4.20876 | 0.789203 | -4.9479 | -1.65466 |
| species | *OTU74000* | -4.20008 | -4.42566 | 0.583955 | -4.9479 | -1.67468 |
| species | *OTU7445* | -3.48074 | -3.39872 | 0.600587 | -5.02378 | -1.94244 |
| species | *OTU7731* | -3.57778 | -3.54452 | 0.719975 | -4.9479 | -1.89456 |
| species | *OTU81797* | -4.21358 | -4.43476 | 0.607295 | -5.02378 | -1.93632 |
| species | *OTU81863* | -4.12111 | -4.37011 | 0.510868 | -4.93233 | -2.26951 |
| species | *OTU82932* | -3.94722 | -4.31572 | 0.679891 | -5.02378 | -2.31232 |
| species | *OTU83309* | -3.75742 | -3.75374 | 0.581932 | -4.84991 | -2.31081 |

| | | | | | | |
|---|---|---|---|---|---|---|
| species | *OTU8371* | -3.76938 | -3.84559 | 0.712584 | -5.02378 | -1.8767 |
| species | *OTU8674* | -2.14747 | -1.91116 | 0.890267 | -4.9479 | -0.78158 |
| species | *OTU8773* | -2.23689 | -2.00781 | 0.779774 | -4.68153 | -1.13829 |
| species | *OTU9283* | -3.50389 | -3.63367 | 0.92119 | -4.84991 | -1.08642 |
| species | *OTU9465* | -3.38508 | -3.32167 | 0.581715 | -4.7489 | -2.17763 |
| species | *OTU9628* | -3.95166 | -4.25842 | 0.642719 | -4.9479 | -2.39398 |
| species | *OTU9952* | -3.23312 | -3.37134 | 1.086413 | -5.02378 | -0.44953 |
| species | *Parabacteroides_OTU3* | -2.75038 | -2.69368 | 0.623876 | -4.41929 | -1.02101 |
| species | *Parabacteroides_OTU4* | -2.51175 | -2.41222 | 0.641986 | -4.28262 | -1.06389 |
| species | *Parabacteroides_OTU8* | -2.20975 | -2.1569 | 0.624908 | -4.30492 | -0.86008 |
| species | *Roseburia_OTU7* | -2.82821 | -2.76805 | 0.476097 | -4.3996 | -1.50323 |
| species | *Ruminococcus lactaris ATCC 29* | -2.446 | -2.41112 | 0.540844 | -4.34139 | -1.06045 |
| species | *Ruminococcus torques ATCC 27* | -2.86667 | -2.73167 | 0.720781 | -4.51092 | -1.45032 |
| species | *Streptococcus suis 05ZYH33* | -3.57729 | -3.60363 | 0.449597 | -4.55945 | -1.99328 |
| species | *Streptophyta_OTU17* | -3.12519 | -3.23411 | 0.823953 | -4.60249 | -0.48468 |
| species | *TM7_genera_incertae_sedis_C* | -3.25658 | -3.21633 | 0.567614 | -4.55823 | -1.61921 |
| species | *TM7_genera_incertae_sedis_C* | -3.06357 | -2.97154 | 0.622619 | -4.51092 | -1.49338 |
| species | *TM7_genera_incertae_sedis_C* | -3.43513 | -3.39141 | 0.565858 | -4.55823 | -1.75789 |
| species | *Turicibacter_OTU30* | -3.10466 | -3.24638 | 0.948272 | -4.64687 | -0.84344 |
| species | *Variovorax paradoxus (T)* | -2.85 | -2.84238 | 0.57944 | -4.25101 | -0.66529 |
| species | *Weissella confusa (T)* | -2.93596 | -2.81152 | 0.62674 | -4.64687 | -1.22192 |
