# Supplementary material for: Host genetics and diet, but not immunoglobulin A expression, converge to shape compositional features of the gut microbiome in an advanced intercross population of mice
Source: Genome Biol. 2014 Dec 17;15(12):552. doi: 10.1186/s13059-014-0552-6 (PMC4290092; doi:10.1186/s13059-014-0552-6)
Supplement: Additional file 5: — Table showing the QTL statistics for the G4 traits showing significant QTLs. [file 13059_2014_552_MOESM5_ESM.pdf]

Additional File 5. QTL statistics for the microbiome traits in the G_4 mouse population.

Trait	Nearest Marker	MMU	Peak Position (Mb)	Naive LOD	GRAIP LOD	CI (Mb)
Helicobacter ganmani (T)	JAX00138460	6	18.6	8.7	4.0*	-40
Lactococcus lactis	JAX00022058	10	106.9	8.2	4.0*	95-111
OTU4622	JAX00005735	1	80.0	9.6	4.0*	58-139
OTU4622	JAX00300375	10	119.4	6.2	3.9	115-123
OTU20360	JAX00258190	1	90.0	8.3	4.0*	70-146
OTU11573	JAX00008045	1	112.7	8.2	4.0*	70-140
OTU11573	JAX00010715	1	149.0	5.8	3.7	143-150
OTU16907	JAX00261568	1	106.6	8.5	4.0*	66-139
OTU41577	JAX00008045	1	112.7	9.0	4.0*	57-152
OTU72899	JAX00240652	1	3.5	7.3	4.0*	-13
	JAX00436582	17	33.2	6.5	4.0*	28-48
OTU390	JAX00431384	17	10.4	9.1	3.9	7-15
OTU42081	JAX00171082	9	49.8	8.6	3.6	45-54
	JAX00431384	17	10.4	7.3	3.6	6-15
OTU12884	JAX00010715	1	148.6	6.1	3.9	143-150
OTU31161	JAX00160567	8	36.6	8.0	3.7	32-45
OTU15599	JAX00008766	1	122.5	7.2	4.0*	104-125
Turicibacter_OTU30	JAX00645933	7	85.9	8.0	4.0*	69-114

LOD exceeding the 95% ($P \leq 0.05$, LOD ≥ 3.9) permutation threshold are denoted by *; other QTL exceeded the 90% ($P \leq 0.1$, LOD ≥ 3.5) threshold.

Confidence intervals (CIs) for QTL positions were obtained using a 1.0 LOD drop in Mb (relative to the GRAIP permuted LOD score).
