# Supplementary material for: Host genetics and diet, but not immunoglobulin A expression, converge to shape compositional features of the gut microbiome in an advanced intercross population of mice
Source: Genome Biol. 2014 Dec 17;15(12):552. doi: 10.1186/s13059-014-0552-6 (PMC4290092; doi:10.1186/s13059-014-0552-6)
Supplement: Additional file 6: — Table showing the basic statistics for the 67 IgA expression traits. [file 13059_2014_552_MOESM6_ESM.pdf]

Additional file 6. Means, standard deviations (SD), and minimum and maximum values of the log of the of the proportion of sequence reads for each IgA taxon. The sample size = 300 in all cases.

IgA	Mean	SD	Minimum	Maximum
B30	-2.101	0.339	-3.298	-1.092
B44	-2.123	0.448	-3.800	-1.156
B45	-1.864	0.378	-3.077	-0.712
B76	-1.617	0.337	-2.556	-0.523
B41	-1.453	0.268	-2.232	-0.629
B88	-1.543	0.472	-3.027	-0.449
B89	-1.383	0.313	-2.396	-0.271
B59	-1.226	0.237	-1.987	-0.598
B178	-2.360	0.359	-3.379	-1.263
B244	-2.281	0.472	-3.743	-1.187
B48	-2.088	0.462	-3.901	-0.901
B57	-1.989	0.383	-3.227	-0.789
B23	-2.322	0.398	-3.692	-1.200
B212	-2.121	0.418	-3.815	-0.824
B11	-2.146	0.519	-4.305	-0.480
B17	-1.814	0.427	-3.828	-0.760
B75	-1.787	0.477	-4.200	-0.482
B37	-1.593	0.549	-3.928	-0.425
B200	-2.421	0.414	-4.074	-1.353
B14	-2.406	0.434	-3.803	-1.138
B80	-2.409	0.485	-3.817	-1.053
B42	-2.228	0.525	-3.572	-0.830
B71	-2.094	0.511	-4.081	-0.879
B172	-2.062	0.448	-3.771	-1.018
B198	-2.553	0.409	-3.713	-1.560
B91	-1.906	0.506	-4.353	-1.069
B72	-1.930	0.612	-4.034	-0.661
B54	-2.323	0.482	-3.558	-0.607
B87	-2.049	0.654	-3.793	-0.305
B67	-2.530	0.495	-4.185	-1.096
B15	-2.503	0.509	-4.208	-1.059
B78	-2.332	0.576	-4.194	-0.821
B93	-1.812	0.746	-4.193	-0.076
B61	-2.046	0.668	-4.150	-0.394
B79	-2.018	0.712	-4.231	0.015
B73	-2.735	0.509	-4.228	-1.345
B43	-2.506	0.641	-4.500	-1.118
B195	-1.974	0.658	-4.456	-0.742
B55	-2.698	0.559	-4.587	-1.370
B50	-2.583	0.656	-4.370	-0.596

B46	-2.022	0.797	-4.664	-0.597
B33	-2.812	0.495	-4.214	-1.089
B92	-2.367	0.747	-4.241	-0.560
B64	-2.753	0.639	-4.401	-1.353
B60	-2.448	0.745	-4.298	-0.594
B82	-2.823	0.559	-4.435	-1.153
B182	-2.911	0.486	-4.266	-1.465
B10	-1.840	0.927	-4.539	-0.450
B40	-2.885	0.551	-4.479	-0.847
B153	-1.938	0.932	-4.884	-0.377
B122	-3.187	0.414	-4.492	-1.943
B234	-3.201	0.492	-4.652	-1.729
B83	-3.192	0.519	-4.573	-1.425
B114	-2.582	0.819	-4.934	-0.601
B237	-3.272	0.535	-4.773	-1.450
B194	-3.209	0.593	-4.585	-1.682
B197	-3.269	0.581	-4.734	-1.606
B217	-2.944	0.693	-4.913	-1.216
B223	-3.096	0.633	-4.810	-1.345
B81	-3.217	0.548	-4.872	-1.850
B47	-3.177	0.623	-4.722	-1.112
B63	-3.119	0.683	-4.770	-1.412
B218	-3.086	0.693	-5.082	-1.567
B189	-3.253	0.577	-4.510	-1.649
B207	-3.065	0.684	-5.061	-1.600
B175	-3.268	0.632	-4.761	-1.518
B196	-3.383	0.582	-4.821	-1.645
