# Supplementary material for: Host genetics and diet, but not immunoglobulin A expression, converge to shape compositional features of the gut microbiome in an advanced intercross population of mice
Source: Genome Biol. 2014 Dec 17;15(12):552. doi: 10.1186/s13059-014-0552-6 (PMC4290092; doi:10.1186/s13059-014-0552-6)
Supplement: Additional file 7: — Table showing the contributions of four variance components to the total variation in the 67 IgA expression traits. [file 13059_2014_552_MOESM7_ESM.pdf]

Additional file 7. Estimates of four random components (cohort, family, parity, and residual) and their proportion of the total variation, for each of the 67 IgA taxa.

	Variance Components				% of Total Variation			
Taxon	Cohort	Family	Parity	Residual	Cohort	Family	Parity	Residual
B30	0.0016	0.0000	0.0177	0.1085	1.26	0.00	13.85	84.89
B44	0.0000	0.0000	0.0176	0.1977	0.00	0.00	8.16	91.84
B45	0.0000	0.0089	0.0088	0.1354	0.00	5.82	5.72	88.46
B76	0.0000	0.0012	0.0090	0.1105	0.00	1.01	7.44	91.55
B41	0.0118	0.0000	0.0113	0.0674	13.07	0.00	12.53	74.39
B88	0.0580	0.0515	0.0000	0.1940	19.12	16.98	0.00	63.90
B89	0.0036	0.0110	0.0050	0.0906	3.28	10.02	4.55	82.16
B59	0.0047	0.0116	0.0000	0.0498	7.17	17.54	0.00	75.29
B178	0.0000	0.0029	0.0064	0.1282	0.00	2.09	4.69	93.22
B244	0.0000	0.0024	0.0182	0.2185	0.00	1.00	7.61	91.39
B48	0.0147	0.0092	0.0018	0.2159	6.10	3.81	0.73	89.36
B57	0.0011	0.0207	0.0000	0.1371	0.68	13.05	0.00	86.26
B23	0.0000	0.0415	0.0000	0.1421	0.00	22.61	0.00	77.39
B212	0.0000	0.0027	0.0066	0.1741	0.00	1.47	3.59	94.94
B11	0.0097	0.0000	0.0000	0.2873	3.25	0.00	0.00	96.75
B17	0.0000	0.0253	0.0193	0.1500	0.00	12.99	9.91	77.09
B75	0.0065	0.0129	0.0038	0.2263	2.61	5.16	1.50	90.72
B37	0.0051	0.0816	0.0156	0.2369	1.51	24.06	4.60	69.83
B200	0.0112	0.0000	0.0000	0.1811	5.84	0.00	0.00	94.16
B14	0.0000	0.0265	0.0000	0.1739	0.00	13.23	0.00	86.77
B80	0.0000	0.0242	0.0000	0.2253	0.00	9.71	0.00	90.29
B42	0.0161	0.0186	0.0360	0.2505	5.00	5.80	11.20	78.00
B71	0.0000	0.0080	0.0172	0.2479	0.00	2.93	6.30	90.77
B172	0.0034	0.0631	0.0000	0.1608	1.50	27.76	0.00	70.74
B198	0.0035	0.0027	0.0000	0.1735	1.97	1.48	0.00	96.55
B91	0.0031	0.0372	0.0253	0.2218	1.09	12.93	8.81	77.18
B72	0.0403	0.1533	0.0000	0.2863	8.39	31.95	0.01	59.66
B54	0.0000	0.0350	0.0000	0.2128	0.00	14.12	0.00	85.88
B87	0.0164	0.1127	0.0380	0.3399	3.23	22.23	7.50	67.04
B67	0.0047	0.0244	0.0000	0.2405	1.74	9.05	0.00	89.21
B15	0.0019	0.0130	0.0186	0.2482	0.68	4.62	6.59	88.11
B78	0.0046	0.0016	0.0294	0.3276	1.26	0.44	8.10	90.21
B93	0.0117	0.1826	0.0420	0.4140	1.81	28.08	6.45	63.66
B61	0.0202	0.1378	0.0520	0.3234	3.79	25.84	9.75	60.62
B79	0.0314	0.1865	0.0320	0.3708	5.05	30.04	5.16	59.74
B73	0.0037	0.0349	0.0000	0.2495	1.29	12.11	0.00	86.60
B43	0.0115	0.0000	0.0329	0.4111	2.53	0.00	7.23	90.24
B195	0.0459	0.0542	0.0036	0.4090	8.95	10.58	0.69	79.78
B55	0.0154	0.0653	0.0360	0.2575	4.11	17.45	9.62	68.82
B50	0.0000	0.0000	0.0133	0.4382	0.00	0.00	2.94	97.06

B46	0.0000	0.2332	0.0146	0.4718	0.00	32.40	2.03	65.56
B33	0.0112	0.0000	0.0187	0.2443	4.07	0.00	6.81	89.12
B92	0.0136	0.1160	0.0192	0.4821	2.16	18.39	3.05	76.41
B64	0.0363	0.0621	0.0040	0.3788	7.54	12.91	0.83	78.72
B60	0.0047	0.1447	0.0587	0.4269	0.74	22.79	9.24	67.23
B82	0.0299	0.0793	0.0000	0.2668	7.95	21.08	0.00	70.97
B182	0.0161	0.0000	0.0020	0.2481	6.04	0.00	0.76	93.20
B10	0.0042	0.3176	0.0000	0.6572	0.43	32.44	0.00	67.13
B40	0.0002	0.0080	0.0066	0.3115	0.06	2.44	2.04	95.46
B153	0.0003	0.2676	0.0000	0.7033	0.03	27.55	0.00	72.42
B122	0.0000	0.0077	0.0080	0.1660	0.00	4.22	4.42	91.37
B234	0.0109	0.0000	0.0376	0.2280	3.94	0.00	13.58	82.48
B83	0.0072	0.0510	0.0000	0.2470	2.36	16.72	0.00	80.92
B114	0.0000	0.1388	0.0692	0.5401	0.00	18.56	9.25	72.19
B237	0.0229	0.0150	0.0002	0.2901	6.98	4.56	0.07	88.40
B194	0.0000	0.0023	0.0000	0.3666	0.00	0.63	0.00	99.37
B197	0.0016	0.0169	0.0000	0.3433	0.44	4.67	0.00	94.90
B217	0.0086	0.0812	0.0083	0.4401	1.61	15.09	1.54	81.77
B223	0.0000	0.1006	0.0000	0.3398	0.00	22.84	0.00	77.16
B81	0.0263	0.0499	0.0087	0.2735	7.33	13.93	2.42	76.32
B47	0.0078	0.0484	0.0000	0.3704	1.83	11.35	0.00	86.82
B63	0.0135	0.1493	0.0006	0.3693	2.54	28.03	0.11	69.32
B218	0.0205	0.1287	0.0005	0.4002	3.73	23.40	0.10	72.76
B189	0.0293	0.0095	0.0000	0.3448	7.65	2.47	0.00	89.88
B207	0.0168	0.1330	0.0000	0.3826	3.16	24.99	0.00	71.86
B175	0.0000	0.0494	0.0370	0.3538	0.00	11.22	8.41	80.37
B196	0.0000	0.0640	0.0000	0.3043	0.00	17.37	0.00	82.63
